# A general framework for subgroup detection via one-step value difference estimation

**DOI:** 10.1111/biom.13711

**Published:** 2022-08-02

**Authors:** Dana Johnson, Wenbin Lu, Marie Davidian

**Affiliations:** 1 United Therapeutics Corp., Research Triangle Park, Durham, North Carolina, USA; 2 Department of Statistics, North Carolina State University, Raleigh, North Carolina, USA

**Keywords:** exceptional law, optimal treatment rule, precision medicine, subgroup test, survival analysis, value function

## Abstract

Recent statistical methodology for precision medicine has focused on either identification of subgroups with enhanced treatment effects or estimating optimal treatment decision rules so that treatment is allocated in a way that maximizes, on average, predefined patient outcomes. Less attention has been given to subgroup testing, which involves evaluation of whether at least a subgroup of the population benefits from an investigative treatment, compared to some control or standard of care. In this work, we propose a general framework for testing for the existence of a subgroup with enhanced treatment effects based on the difference of the estimated value functions under an estimated optimal treatment regime and a fixed regime that assigns everyone to the same treatment. Our proposed test does not require specification of the parametric form of the subgroup and allows heterogeneous treatment effects within the subgroup. The test applies to cases when the outcome of interest is either a time-to-event or a (uncensored) scalar, and is valid at the exceptional law. To demonstrate the empirical performance of the proposed test, we study the type I error and power of the test statistics in simulations and also apply our test to data from a Phase III trial in patients with hematological malignancies.

## 1 | INTRODUCTION

Consider the setting in which an active treatment and a control are to be compared on the basis of some outcome—which may be continuous, discrete, or a possibly censored time-to-event. Due to patient treatment effect heterogeneity, it is possible that a subgroup of patients benefit from the active treatment, but the overall estimated treatment effect is nonsignificant or borderline significant. When this happens, one may want to “salvage” the treatment by testing whether at least some subgroup benefits from treatment, and, if the null hypothesis of no benefit is rejected, identifying the subgroup(s) with an enhanced treatment effect (Lipkovich et al., 2017). For example, Lipkovich et al. (2017) describe a Phase III clinical trial in patients with hematological malignancies that resulted in a hazard ratio (treatment vs. control) for overall survival that was borderline significant (one-sided p=0.0367). Due to this borderline result, the sponsor decided to search for subgroups with improved benefit/risk ratios. To handle situations similar to those described above, statistical methods from the precision medicine literature are often applied. This literature mainly focuses on one of two things: estimation of an optimal treatment regime, or identification of subgroups with enhanced treatment effects. Various parametric or nonparametric methods have been developed for the estimation of optimal treatment regimes with both uncensored outcomes (eg, Watkins and Dayan, 1992; Murphy, 2003; Zhang et al., 2012a, b; Zhao et al., 2012) and censored time-to-event outcomes (eg, Bai et al., 2017; Goldberg and Kosorok, 2012; Jiang et al., 2017; Zhao et al., 2015). Likewise, intensive research has been done for identifying subgroups with enhanced/differential treatment effects (eg, Cai et al., 2010; Foster et al., 2011; Lipkovich et al., 2011; Li et al., 2019; Wang et al., 2019; Zhao et al., 2013). However, less work has been done in the area of subgroup testing. Here the focus is to test whether or not one treatment is better than the other for at least some subgroup of the population. As argued by Shi et al. (2020), this is a foundational question that needs to be answered prior to either estimating an optimal treatment regime or searching for subgroups with enhanced treatment effects. Existing subgroup testing literature includes parametric mixture models (Shen and He, 2015; Wu et al., 2016) and change-plane approaches (Fan et al., 2017; Kang et al., 2017; Wei and Kosorok, 2018). Some limitations of these approaches are that the subgroup is assumed to have some parametric form, such as defined by a linear function, and/or the treatment effect is assumed to be constant within the subgroup. Such assumptions may be restrictive in practical applications. These challenges motivate us to develop a general framework for testing for the existence of a subgroup with enhanced treatment effects, which does not require specification of the form of the subgroup and allows heterogeneous treatment effects within the subgroup.

Our proposed test statistics are built based on estimators of the value difference between an estimated optimal regime and a fixed regime that assigns everyone to the same treatment. Both uncensored outcomes and censored time-to-event outcomes are considered. To derive the asymptotic distributions of the test statistics under the null hypothesis, we adapt the one-step estimation procedure proposed by Luedtke and van der Laan (2016, LL16 henceforth), which can lead to valid inference even under the exceptional law, that is, when the treatment effect of individual patients can be zero with a positive probability. In addition, within the one-step estimation framework, we consider two schemes to partition observations into smaller chunks of data and show that one particular partition can enhance the power of the considered test statistic under certain settings. In Section 2, we introduce notation and assumptions. In Section 3, we propose several value difference estimators for both uncensored and censored outcomes. In Section 4, we introduce our test procedure. We study the performance of the proposed test statistics via extensive simulations in Section 5 and apply the proposed test to the Phase III trial data described in Lipkovich et al. (2017) in Section 6.

## 2 | STATISTICAL FRAMEWORK FOR SUBGROUP TESTING

We first introduce the framework in the case of an uncensored outcome. Let X be a p×1 vector of baseline covariates taking values in 𝒳. Let Y be an observed, continuous outcome coded so larger values are preferred, and let A be a binary treatment indicator, where A=0 corresponds to control/standard of care, and A=1 corresponds to the active treatment. The observed data consist of independent and identically distributed (iid) copies of O={X,A,Y}, indexed by i=1,…,n. Let $Y^{*}(a)$ be the potential outcome (Rubin, 1974) a patient could experience if he/she were to receive treatment option a,a=0,1. We assume that

(1) $$E\{Y^{*}(a)|X\}=C_{0}\left(X\right)+a\tau \left(X\right),$$

where $C_{0}(X)$ is the expected conditional outcome if the control were assigned to all patients with X in the population, and $\tau (X)=E\{Y^{*}(1)|X\}-E\{Y^{*}(0)|X\}$ is the conditional average treatment effect (CATE).

We assume the stable unit treatment value assumption (SUTVA) holds, which implies that $Y=Y^{*}(A)$. In addition, we assume both the positivity assumption and the no unmeasured confounders assumption hold, which state that pr⁡(A=a∣X=x)>0 for all x∈𝒳 and that $\{Y^{*}(0),Y^{*}(1)\}⫫A|X$, respectively, where ⫫ means “independent” and the notation ⋅⫫⋅∣X refers to conditional independence given X. Under these assumptions, it can be shown that τ(X)=E(Y∣X,A=1)-E(Y∣X,A=0).

We are interested in testing whether or not the active treatment is beneficial, relative to control, for at least some patients in the population. Therefore, we are interested in testing

(2) $$H_{0}\;:\;\tau (x)\leq 0\;\text{for}\;\text{all}\;x\in 𝒳\;\text{vs.}\;H_{1}:\exists {𝒳}_{1}\subset 𝒳\;\text{with}\;pr\left(X\in {𝒳}_{1}\right)>0\;\;\text{such}\;\text{that}\;\tau \left(x\right)>0\;\text{for}\;\text{all}\;x\in {𝒳}_{1};$$

where, importantly, we allow a portion of the population to not experience a treatment effect, that is, we allow pr⁡{τ(X)=0}>0. This is referred to as the exceptional law. Let d be a treatment rule that maps information in X to a treatment decision in {0, 1}. The potential outcome under d is defined as $Y^{*}\{d(X)\}=Y^{*}(1)1\{d(X)=1\}+Y^{*}(0)1\{d(X)=0\}$, where 1(⋅) is the indicator function with 1(B)=1 if the event is true and = 0 otherwise. The value of a treatment rule is defined as $V(d)=E[Y^{*}\{d(X)\}]$, and an optimal treatment rule, $d^{opt}\equiv d^{opt}(X)=arg{max}_{d}\;V(d)$. Under our considered model and assumptions, the optimal treatment rule is given by $d^{\text{opt}}(X)=1\{\tau (X)>0\}$. Note that when τ(X)=0 the defined optimal rule selects the control for unique representation. Let d(X)≡0 denote the regime that gives everyone treatment 0. Define $\Psi \left(d^{\text{opt}}\right)=V\left(d^{\text{opt}}\right)-V(0)$, which is the value difference under the optimal rule and the fixed regime d(X)≡0. It can be shown that $\Psi \left(d^{\text{opt}}\right)=0$ under $H_{0}$, while it is positive under $H_{1}$. This motivates us to use estimators of the value difference as test statistics for testing the considered hypothesis in (2). If the outcome of interest is a right-censored time-to-event, the above framework can be modified. The observed data become O={X,A,U,Δ}, where T is the event time of interest, C is the censoring time, U=min(T,C), and Δ=1(T≤C). The definition of the CATE changes from a difference in conditional means to a difference in conditional restricted mean survival times. Let $T^{*}(a)$ denote the potential time to event had treatment a been administered, and let L be such that pr⁡(U≥L)>0. The restricted mean survival time (RMST) under treatment a, conditional on X, is defined as $E[min\{T^{*}(a),L\}|X]=\int_{0}^{L}S^{*a}(t|X)dt$, where $S^{*a}(t|X)=P(T^{*}(a)\geq t|X)$ is the conditional survival function under treatment a. Then, the CATE is $\tau (X)=E[min\{T^{*}(1),L\}|X]-C_{0}(X)$, where $C_{0}(X)=E[min\{T^{*}(0),L\}|X]$. In addition to the positivity assumption and extensions of the SUTVA and no unmeasured confounders assumption to time-to-event data, we assume that censoring is noninformative, that is, that $C⫫\{T^{*}(0),T^{*}(1)\}|X$. Under these four assumptions, τ(X) can be identified as a function of the observed data (see Web Appendix A in the Supporting Information). We consider the same hypothesis as defined in (2), but the value function is now defined based on the restricted mean survival time, that is, $V(d)=E(min[T^{*}\{d(X)\},L])$.

## 3 | VALUE DIFFERENCE ESTIMATORS

### 3.1 | Value difference estimators for an uncensored outcome

To construct estimators for the value difference $\Psi \left(d^{\text{opt}}\right)$, we use the inverse propensity score weighted estimators proposed by Zhang et al. (2012b) for the value under the optimal rule and the fixed regime d≡0, respectively. Specifically, define

(3) $$S_{IPW}\left(O_{i}\right)\;=\left\{\frac{1\left[A_{i}=1\left\{\tau \left(X_{i}\right)>0\right\}\right]}{\pi_{A_{i}}\left(X_{i}\right)}Y_{i}\right.\;\;-\left(\frac{1\left[A_{i}=1\left\{\tau \left(X_{i}\right)>0\right\}\right]}{\pi_{A_{i}}\left(X_{i}\right)}-1\right)\;\;\left.\times \left[C_{0}\left(X_{i}\right)+\tau \left(X_{i}\right)1\left\{\tau \left(X_{i}\right)>0\right\}\right]\right\}-\left\{\frac{1\left(A_{i}=0\right)}{1-\pi \left(X_{i}\right)}Y_{i}\right\},$$

(4) $$S_{AIPW}\left(O_{i}\right)=S_{IPW}\left(O_{i}\right)+\left\{\frac{1\left(A_{i}=0\right)}{1-\pi \left(X_{i}\right)}-1\right\}C_{0}\left(X_{i}\right),$$

and ${\hat{\Psi }}_{•}\equiv {\hat{\Psi }}_{•}(O)=n^{-1}\sum_{i=1}^{n}\;S_{•}\left(O_{i}\right)$, where π(X)=pr⁡(A=1∣X) is the propensity score, $\pi_{A}(X)=A\pi (X)+(1-A)\{1-\pi (X)\}$, and $S_{•}$ serves as a placeholder for either $S_{\text{IPW}}$ or $S_{\text{AIPW}}$. In both ${\hat{\Psi }}_{\text{IPW}}$ and ${\hat{\Psi }}_{\text{AIPW}}$ the estimator for $V\left(d^{opt}\right)$, which corresponds to the first three terms of (3) and the first term of (4), is an augmented inverse probability weighted (AIPW) estimator, whereas the estimator for V(0), which corresponds to the last term in (3) and (4), is an inverse probability weighted (IPW) estimator in ${\hat{\Psi }}_{\text{IPW}}$, but an augmented inverse probability weighted estimator in ${\hat{\Psi }}_{\text{AIPW}}$. When constructing the value difference estimators ${\hat{\Psi }}_{\text{IPW}}$ and ${\hat{\Psi }}_{\text{AIPW}}$, the functions $\pi ,\;C_{0}$, and τ are estimated from the data. The details will be given in the next section.

#### *Remark* 1.

Inspection of (4) reveals that ${\hat{\Psi }}_{\text{AIPW}}$ is exactly equal to 0 whenever $1\left\{\tau \left(X_{i}\right)>0\right\}=0$ for all i. In the regular setting, that is, pr⁡{τ(X)=0}=0, we have τ(X)<0 with probability 1 under $H_{0}$. Therefore, ${\hat{\Psi }}_{\text{AIPW}}$ under $H_{0}$ would be exactly equal to 0 asymptotically because $\hat{\tau }\left(X_{i}\right)$ will become negative for all i as n goes to infinity, as long as $\hat{\tau }$ is a consistent estimator of τ. This implies that the limiting null distribution of ${\hat{\Psi }}_{\text{AIPW}}$ will be degenerate under the regular setting. On the contrary, the limiting null distribution of ${\hat{\Psi }}_{\text{IPW}}$ will still be normal because the IPW estimator of V(0) used in ${\hat{\Psi }}_{\text{IPW}}$ does not exactly cancel with the AIPW estimator of $V\left(d^{opt}\right)$ under the null.

### 3.2 | Value difference estimators for censored outcome

Let $U_{i}^{L}=min\left(U_{i},L\right)$ and $\Delta_{i}^{L}=1\left(U_{i}^{L}\leq C_{i}\right)=\Delta_{i}+(1-\left.\Delta_{i}\right)1\left(U_{i}\geq L\right)$. Define

(5) $$S_{IPW}^{S}\left(O_{i}\right)\;=\left(\frac{\Delta_{i}^{L}1\left[A_{i}=1\left\{\tau \left(X_{i}\right)>0\right\}\right]}{K_{c}\left(U_{i}^{L}|X_{i},A_{i}\right)\pi_{A_{i}}\left(X_{i}\right)}U_{i}^{L}\right.\;\;\left.-\left(\frac{1\left[A_{i}=1\left\{\tau \left(X_{i}\right)>0\right\}\right]}{\pi_{A_{i}}\left(X_{i}\right)}-1\right)\left[\zeta_{i}C_{0}\left(X_{i}\right)+\tau \left(X_{i}\right)1\left\{\tau \left(X_{i}\right)>0\right\}\right]\right)\;\;-\left[\frac{\Delta_{i}^{L}1\left(A_{i}=0\right)}{K_{c}\left(U_{i}^{L}|X_{i},A_{i}\right)\left\{1-\pi \left(X_{i}\right)\right\}}U_{i}^{L}\right],$$

(6) $$S_{AIPW}^{S}\left(O_{i}\right)=S_{IPW}^{S}\left(O_{i}\right)+\left\{\frac{1\left(A_{i}=0\right)}{1-\pi \left(X_{i}\right)}-1\right\}C_{0}\left(X_{i}\right),$$

(7) $$S_{CAIPW}^{S}\left(O_{i}\right)\;=S_{AIPW}^{S}\left(O_{i}\right)+\frac{1\left[A_{i}=1\left\{\tau \left(X_{i}\right)>0\right\}\right]}{\pi_{A_{i}}\left(X_{i}\right)}\int_{0}^{L}\;\frac{dM_{c}\left(r|X_{i},A_{i}\right)}{K_{c}\left(r|X_{i},A_{i}\right)}m\left(r|X_{i},A_{i}\right)\;\;-\frac{1\left(A_{i}=0\right)}{1-\pi \left(X_{i}\right)}\int_{0}^{L}\;\frac{dM_{c}\left(r|X_{i},A_{i}\right)}{K_{c}\left(r|X_{i},A_{i}\right)}m\left(r|X_{i},A_{i}\right),$$

and $\;{\hat{\Psi }}_{•}^{S}\equiv {\hat{\Psi }}_{•}^{S}(O)=n^{-1}\sum_{i=1}^{n}\;S_{•}^{S}\left(O_{i}\right),\;$ where $\;K_{c}(t|\left.X_{i},A_{i}\right)=pr\left(C_{i}\geq t|X_{i},A_{i}\right)$ is the conditional survival function of censoring times, $\Lambda_{c}\left(r|X_{i},A_{i}\right)$ is the associated conditional cumulative hazard function, $\;dM_{c}\left(r|X_{i},A_{i}\right)=dN_{c}(r)-Y(r)d\Lambda_{c}\left(r|X_{i},A_{i}\right),\;N_{c}(r)=1\left(U_{i}\leq r,\Delta_{i}=0\right)$ is the censoring counting process, $Y(r)=1\left(U_{i}\geq r\right)$ is the at risk process, $m\left(r|X_{i},A_{i}\right)=E\left\{min(T,L)|T\geq r,X_{i},A_{i}\right\}$, and $\zeta_{i}$ equals 1 in (5) and is a mean one perturbation term in (6) and (7); see Remark 2. We use $S_{•}^{S}$ as a placeholder for $S_{IPW}^{S},$ $S_{AIPW}^{S}$, or $S_{CAIPW}^{S}$. The estimator in (7) is called a “CAIPW” estimator because it is an AIPW estimator with additional censoring augmentation terms (the “C” refers to censoring). Specifically, the second and third terms on the right-hand side of (7) are censoring augmentation terms under $d^{\text{opt}}$ and d(X)≡0, respectively, which can improve the efficiency of the value difference estimator by recovering information on censored but regime-consistent patients. It can be shown that the CAIPW estimator is the locally efficient estimator for the value difference (Bai et al., 2013) and has a desirable double robustness property—meaning if either the propensity model and the model for the censoring distribution are correctly specified, or if the conditional distribution of $T_{i}|X_{i},A_{i}$ is correctly specified, ${\hat{\Psi }}_{\text{CAIPW}}^{S}$ is a consistent estimator for the value difference. If the data come from a randomized trial, so that π and the censoring distribution can be correctly specified, then all of ${\hat{\Psi }}_{IPW}^{S},$ ${\hat{\Psi }}_{AIPW}^{S}$, and ${\hat{\Psi }}_{CAIPW}^{S}$ are consistent estimators for $\Psi \left(d^{\text{opt}}\right)$ (Bai et al., 2017).

#### *Remark* 2.

For reasons described in Web Appendix B of the Supporting Information, $\zeta_{i}$ needs to be included in (6) and (7) because without $\zeta_{i}$ it is likely for ${\hat{\Psi }}_{j,\text{AIPW}}^{S}(C_{j},{𝒪}_{j-1}^{*})$ and ${\hat{\Psi }}_{j,\text{CAIPW}}^{S}({𝒞}_{j},{𝒪}_{j-1}^{*})$ (described in Section 4.2) to be exactly equal to zero even at the exceptional law—unlike for an uncensored outcome, for which this is a concern only in the regular setting.

## 4 | TEST PROCEDURES

### 4.1 | Estimation of nuisance functions

The value difference estimators presented in Sections 3.1 and 3.2 are not directly useful because $\tau (X),C_{0}(X),\pi (X),K_{C}\left(r|X_{i},A_{i}\right),dM_{c}\left(r|X_{i},A_{i}\right)$, and $m\left(r|X_{i},A_{i}\right)$ are usually unknown functions. For an uncensored outcome, we propose estimating $C_{0}(X)$ and τ(X) using random forests (Breiman, 2001). Specifically, we use the counterfactual synthetic random forest approach described by Lu et al. (2018). Refer to Web Appendix C for the justification for using this approach and for details on how this estimator is computed. While choosing a nonparametric method places less assumptions on the form of $C_{0}(X)$ and τ(X), a concern with using these methods is the “curse of dimensionality.” Random forests tend to be more robust to the curse of dimensionality (can handle larger p) than, say, kernel regression. For a censored outcome, we propose estimating $C_{0}(X)$ and τ(X) using random survival forests (Ishwaran et al., 2008). Refer to Web Appendix D for details. We estimate $K_{c}(t|X,A)$ by fitting a Cox proportional hazards model with U as the observed time and 1-Δ as the status. The censoring augmentation term (the part that is an integral), which is a function of nuisance functions, can be rewritten as

(8) $$\int_{0}^{L}\frac{dM_{c}\left(r|X_{i},A_{i}\right)}{K_{c}\left(r|X_{i},A_{i}\right)}m\left(r|X_{i},A_{i}\right)=\;\;\frac{(1-\Delta_{i}^{L})m\left(U_{i}^{L}|X_{i},A_{i}\right)}{K_{c}\left(U_{i}^{L}|X_{i},A_{i}\right)}-\int_{0}^{U_{i}^{L}}\frac{d\Lambda_{c}\left(r|X_{i},A_{i}\right)}{K_{c}\left(r|X_{i},A_{i}\right)}m\left(r|X_{i},A_{i}\right).$$

Refer to Web Appendix E for the proof. In (8), the estimator for the conditional cumulative censoring hazard, which we denote ${\hat{\Lambda }}_{c}(r|X,A)$, is obtained from the same Cox proportional hazards model used to estimate $K_{c}(t|X,A)$. Finally, as discussed in Chapter 8 of Tsiatis et al. (2020), m(r∣X,A) can be written as

(9) $$m(r|X,A)=\int_{r}^{L}\;z\left\{\frac{-dS\left(z|X_{i},A_{i}\right)}{S\left(r|X_{i},A_{i}\right)}\right\}+L\frac{S\left(r|X_{i},A_{i}\right)}{S\left(L|X_{i},A_{i}\right)}.$$

Thus, an estimator for $m\left(r|X_{i},A_{i}\right),$ $\overset{ˆ}{m}\left(r|X_{i},A_{i}\right)$, is obtained by replacing S(⋅∣X,A) in (9) with the random survival forest estimator $\hat{S}(\cdot |X,A)$. For both uncensored and censored outcomes, we estimate the propensity score using logistic regression.

### 4.2 | One-step framework

We replace the nuisance functions in the expressions for the value difference estimators in Section 3 with the estimators described in Section 4.1, and we follow the one-step estimation framework in LL16 to handle the nonregularity resulting from estimating $d^{\text{opt}}$ under the exceptional law. We describe the one-step framework and associated data partitioning methods (Section 4.3) in the censored outcome context. The steps and notation for the uncensored outcome setting are analogous and are given in the Web Appendix F. We first estimate the nuisance functions using a “chunk” of $l_{n}$ observations. We partition the remaining data into $r_{n}=\left(n-l_{n}\right)/m$ chunks of size m, where m≥1, and $l_{n}$ is chosen such that $\left(n-l_{n}\right)/m\to \infty$ as n→∞. Let ${𝒞}_{j}=\left\{O_{i}\;:O_{i}\in j\right.th\;data\;chunk\}$, so that each ${𝒞}_{j}$ for $j=1,\ldots ,r_{n}$ contains m observations. We also let ${𝒪}_{j}^{*}=\left\{O_{i}\right.\;:\left.O_{i}\in {⋃}_{k=0}^{j}\;c_{k}\right\}$, where ${𝒞}_{0}\equiv {𝒪}_{0}^{*}$ corresponds to all observations in the initial data chunk of size $l_{n}$. Refer to Web Figure 1. Now define

(10) $$S_{j,CAIPW}^{S}(O_{i},{𝒪}_{j-1}^{*})=S_{j,AIPW}^{S}(O_{i},{𝒪}_{j-1}^{*})+\frac{1\left[A_{i}=1\left\{{\overset{ˆ}{\tau }}_{j}\left(X_{i}\right)>0\right\}\right]}{{\overset{ˆ}{\pi }}_{j,A_{i}}\left(X_{i}\right)}\;\;\times \int_{0}^{L}\frac{d{\hat{M}}_{j,c}\left(r|X_{i},A_{i}\right)}{{\hat{K}}_{j,c}\left(r|X_{i},A_{i}\right)}{\hat{m}}_{j}\left(r|X_{i},A_{i}\right)-\frac{1\left(A_{i}=0\right)}{1-{\hat{\pi }}_{j}\left(X_{i}\right)}\int_{0}^{L}\frac{d{\hat{M}}_{j,c}\left(r|X_{i},A_{i}\right)}{{\hat{K}}_{j,c}\left(r|X_{i},A_{i}\right)}{\hat{m}}_{j}\left(r|X_{i},A_{i}\right),$$

and ${\overset{ˆ}{\Psi }}_{j,•}^{S}({𝒞}_{j},{𝒪}_{j-1}^{*})=m^{-1}\sum_{c_{j}}\;S_{j,•}^{S}(O_{i},{𝒪}_{j-1}^{*})$, where ${\overset{ˆ}{\pi }}_{j}\left(X\right),{\overset{ˆ}{\pi }}_{j,A}\left(X\right),{\hat{\tau }}_{j}\left(X\right),\;{\hat{C}}_{j,0}\left(X\right),\;{\hat{K}}_{c}\left(r|X_{i},A_{i}\right),\;d{\hat{M}}_{c}\left(r|X_{i},A_{i}\right)$, and $\hat{m}\left(r|X_{i},A_{i}\right)$ are estimators for $\pi (X),\pi_{A}(X),\tau (X),C_{0}(X),K_{c}\left(r|X_{i},A_{i}\right),dM_{c}\left(r|X_{i},A_{i}\right)$, and $m\left(r|X_{i},A_{i}\right)$, respectively, based on the “historical” data in ${𝒪}_{j-1}^{*}$. At step j, these estimators are known, fixed functions. For brevity, expressions for $S_{j,IPW}^{S}(O_{i},{𝒪}_{j-1}^{*})$ and $S_{j,AIPW}^{S}(O_{i},{𝒪}_{j-1}^{*})$ are not given here, but they are analogous to (10).

We consider the test statistic

(11) $$\begin{array}{l} T_{\cdot ,\text{SBT}}^{S}=r_{n}^{-1/2}\sum_{j=1}^{r_{n}}{\hat{\sigma }}_{j}^{-1}{\hat{\Psi }}_{j,\cdot }^{S}\left({𝒞}_{j},{𝒪}_{j-1}^{*}\right)=r_{n}^{-1/2}\left(\sum_{j=1}^{r_{n}}{\hat{\sigma }}_{j}^{-1}\right){\hat{\Psi }}^{S}(O),\\ {\hat{\Psi }}^{S}(O)=\frac{\sum_{j=1}^{r_{n}}{\hat{\sigma }}_{j}^{-1}{\hat{\Psi }}_{j,\cdot }^{S}\left({𝒞}_{j},{𝒪}_{j-1}^{*}\right)}{\sum_{j=1}^{r_{n}}{\hat{\sigma }}_{j}^{-1}}. \end{array}$$

Here, ${\hat{\Psi }}^{S}(O)$ is a weighted average of the chunk-specific value difference estimators, so that ${\hat{\Psi }}^{S}(O)$ is an estimator for $\Psi \left(d^{opt}\right)$. The ${\hat{\sigma }}_{j}$ are estimators for the standard deviation of ${\hat{\Psi }}_{j,•}^{S}({𝒞}_{j},{𝒪}_{j-1}^{*})$ given ${𝒪}_{j-1}^{*}$ (see Section 4.3). The asymptotic distribution of ${\hat{\Psi }}^{S}(O)$ can be derived using conditions similar to (C1)—(C5) in LL16. Thus, the rejection region for an asymptotically valid, α level test is $T_{•,\;\text{SBT}}>z_{1-\alpha }$, where $z_{1-\alpha }$ denotes the 1-α quantile of the standard normal distribution. Details on how random forests may achieve the convergence rate required by (C4) and (C5) of LL16 are provided in the Web Appendix G.

### 4.3 | Data chunking approaches

There are two ways one might partition the observed data into chunks. One way is to randomly assign observations to a chunk without paying attention to the treatment label, which is the approach in LL16 referred to here as the sequential, blinded-treatment (SBT) approach. Another option is to assign observations to chunks such that the proportion of treatment observations in each chunk preserves the marginal probability of treatment, pr⁡(A=1), in the study. For example, consider a randomized trial where π=0.4 is known, and we set the chunk size m=10. Then observations are first allocated to ${𝒞}_{1}$ through ${𝒞}_{r_{n}}$ such that each of these chunks contains four treatment observations and six control observations. The remaining $l_{n}$ observations are assigned to ${𝒞}_{0}$; although the proportion of treatment observations in ${𝒞}_{0}$ may not equal π, it should be close in large samples. We call this second approach the sequential, averaged-propensity matching (SAP-match) approach. For data from an observational study, the SAP-match approach can be implemented by replacing π with the sample proportion of treatment observations, $\overset{ˆ}{p}$. Thus, for each value difference estimator, there exist two versions of the test statistic—the SBT version and the SAP-match version. For time-to-event outcomes, we use ${\hat{\Psi }}_{j,•}^{S,m\pi }({𝒞}_{j},{𝒪}_{j-1}^{*})$ to denote the SAP-match estimator, whereas ${\hat{\Psi }}_{j,•}^{S}({𝒞}_{j},{𝒪}_{j-1}^{*})$ denotes the SBT estimator. The form of the test statistic in (11), under the SAP-match chunking method, is $T_{•,\text{SAP-match}}^{S}=r_{n}^{-1/2}\sum_{j=1}^{r_{n}}\;{\hat{\sigma }}_{j,m\pi }^{-1}{\hat{\Psi }}_{j,•}^{S,m\pi }({𝒞}_{j},{𝒪}_{j-1}^{*})$, where ${\hat{\sigma }}_{j,m\pi }$ is an estimator for $var{\left\{{\hat{\Psi }}_{j,•}^{S,m\pi }({𝒞}_{j},{𝒪}_{j-1}^{*})|{𝒪}_{j-1}^{*}\right\}}^{1/2}$ formed using only the data in $O_{j-1}^{*}$. Notation for uncensored outcomes is analogous.

When π is known, it is shown in Web Appendix H that $E\{{\hat{\Psi }}_{j,•}^{S,m\pi }({𝒞}_{j},{𝒪}_{j-1}^{*})|{𝒪}_{j-1}^{*}\}=E\{{\hat{\Psi }}_{j,•}^{S}({𝒞}_{j},{𝒪}_{j-1}^{*})|{𝒪}_{j-1}^{*}\}$, so that ${\hat{\Psi }}_{j,•}^{S,m\pi }$ is also a consistent estimator for $\Psi ({\hat{d}}_{j}^{opt})$, where ${\hat{d}}_{j}^{\text{opt}}$ is a fixed estimator for the optimal rule. A similar result holds in the uncensored outcome setting.

When observations are randomly allocated to chunks, which is the case under the SBT approach, $\sigma_{j}$ can be estimated as described in LL16. Specifically, taking $\left|{𝒪}_{j-1}^{*}\right|$ to represent the number of elements in ${𝒪}_{j-1}^{*}$, let

(12) $${\hat{\sigma }}_{j}^{2}=\max\left(q_{j},m^{-1}\left[{\left|{𝒪}_{j-1}^{*}\right|}^{-1}\sum_{O_{i}\in {𝒪}_{j-1}^{*}}{\left\{S_{j,•}^{S}\left(O_{i},{𝒪}_{j-1}^{*}\right)\right\}}^{2}\right.\right.\;\;\left.\left.-{\left\{{\left|{𝒪}_{j-1}^{*}\right|}^{-1}\sum_{O_{i}\in {𝒪}_{j-1}^{*}}S_{j,•}^{S}\left(O_{i},{𝒪}_{j-1}^{*}\right)\right\}}^{2}\right]\right)$$

for any $q_{j}\to 0$, as j→∞, which adapts (14) of LL16 to our setting. The sequence $q_{j}$ ensures that ${\hat{\sigma }}_{j}^{-1}$ is finite for all j. It can depend on both j and $S_{j,•}^{S}(O_{i},{𝒪}_{j-1}^{*})$, for $O_{i}\in {𝒪}_{j-1}^{*}$. When π is known, the variance of ${\hat{\Psi }}_{j,•}^{S,m\pi }({𝒞}_{j},{𝒪}_{j-1}^{*})$, given ${𝒪}_{j-1}^{*}$, is

(13) $$var\left\{{\hat{\Psi }}_{j,•}^{S,m\pi }({𝒞}_{j},{𝒪}_{j-1}^{*})|{𝒪}_{j-1}^{*}\right\}\;=m^{-1}\left[\pi var\left\{S_{j,•}^{S}(O_{i},{𝒪}_{j-1}^{*})|{𝒪}_{j-1}^{*},A_{i}=1\right\}\right.\;\;\left.+(1-\pi )var\left\{S_{j,•}^{S}(O_{i},{𝒪}_{j-1}^{*})|{𝒪}_{j-1}^{*},A_{i}=0\right\}\right],$$

which naturally suggests that we can estimate $\sigma_{j,m\pi }$ by the square root of

(14) $${\hat{\sigma }}_{j,m\pi }^{2}=\text{max}\left\{q_{j},m^{-1}\left(\pi \left[{\left|{𝒪}_{1,j-1}^{\text{*}}\right|}^{-1}\sum_{o_{i}\in {𝒪}_{1,j-1}^{\text{*}}}{\left\{S_{j,•}^{S}\left(O_{i},{𝒪}_{j-1}^{\text{*}}\right)\right\}}^{2}-\left\{{\left|{𝒪}_{1,j-1}^{\text{*}}\right|}^{-1}\right.\right.\right.\right.\;\left.{\left.\times \sum_{O_{i}\in {𝒪}_{1,j-1}^{\text{*}}}S_{j,•}^{S}\left(O_{i},{𝒪}_{j-1}^{\text{*}}\right)\right\}}^{2}\right]+\left(1-\pi \right)\left[{\left|{𝒪}_{0,j-1}^{\text{*}}\right|}^{-1}\sum_{O_{i}\in {𝒪}_{0,j-1}^{\text{*}}}{\left\{S_{j,•}^{S}\left(O_{i},{𝒪}_{j-1}^{\text{*}}\right)\right\}}^{2}\right.\;\left.\left.\left.-{\left\{{\left|{𝒪}_{0,j-1}^{\text{*}}\right|}^{-1}\sum_{O_{i}\in {𝒪}_{0,j-1}^{\text{*}}}S_{j,•}^{S}\left(O_{i},{𝒪}_{j-1}^{\text{*}}\right)\right\}}^{2}\right]\right)\right\}$$

for any $q_{j}\to 0$, as j→∞. In the above, ${𝒪}_{0,j-1}^{*}$ and ${𝒪}_{1,j-1}^{*}$, respectively, denote the sets of observations with A=0 and A=1 that are in the historical data ${𝒪}_{j-1}^{*}$. When π is unknown, $\overset{ˆ}{p}=n^{-1}\sum_{i=1}^{n}\;A_{i}$ can be substituted for π in (14). Theorem 1 shows that ${\hat{\sigma }}_{j}^{2}$ and ${\overset{ˆ}{\sigma }}_{j,m\pi }^{2}$ cannot be used interchangeably; the proof is given in Web Appendix I. Similar arguments can be used to show this relationship also holds for the uncensored outcome setting.

#### Theorem 1.

Under the conditions given in Web Appendix I,

$$var\left\{{\hat{\Psi }}_{j,•}^{S}({𝒞}_{j},{𝒪}_{j-1}^{*})|{𝒪}_{j-1}^{*}\right\}\geq var\left\{{\hat{\Psi }}_{j,•}^{S,m\pi }({𝒞}_{j},{𝒪}_{j-1}^{*})|{𝒪}_{j-1}^{*}\right\}.$$

According to Theorem 1, using the SAP-match approach to construct the data chunks may give the proposed test more power to detect departures from $H_{0}$ than using the SBT approach. The simulations in Section 5.1 show that this power difference is remarkable in the randomized controlled trial setting with (SBT/SAP-match) value difference estimators based on $S_{j,IPW}(O_{i},{𝒪}_{j-1}^{*})$; however, when (SBT/SAP-match) value difference estimators are based on $S_{j\text{,AIPW}}(O_{i},{𝒪}_{j-1}^{*})$, the difference in power is indistinguishable across chunking method. Perhaps this is because $m^{-1}var\left[E\{S_{j,AIPW}(O_{i},{𝒪}_{j-1}^{*})|{𝒪}_{j-1}^{*},A_{i}\}|{𝒪}_{j-1}^{*}\right]$, which appears in the proof of Theorem 1, is sufficiently small under our choice of m=10.

## 5 | SIMULATION STUDIES

### 5.1 | Real-valued, uncensored health outcome

We evaluate the performance of the four versions of the proposed test statistic ($T_{IPW,\text{SAP-match}},\;T_{AIPW,SAP-match},\;T_{IPW,SBT}$, and $T_{AIPW,SBT}$) by generating data from three models of the following form: $Y_{i}=C_{0}\left(X_{i}\right)+A_{i}\tau \left(X_{i}\right)+\varepsilon_{i},\;\varepsilon_{i}$ are iid N(0,0.25). For all three models, $A_{i}$ are Bernoulli with mean π for randomized study simulations that used the SBT approach; $\sum_{i=1}^{n}\;A_{i}=n\pi$ for randomized study simulations that used the SAP-match approach; and Bernoulli with mean $\pi \left(X_{i}\right)$ for all observational data simulations. Across the three models, the proportion of the population with a treatment effect of exactly zero ranged from around 0.26–0.89. Each simulation involved 500 Monte Carlo data sets with n=600 or 1000. In all simulations, $l_{n}=n/2$ and m=10. We defined c to be the treatment effect among the patients who do not have a treatment effect of 0. When $c\leq 0,H_{0}$ is true; when $c>0,H_{1}$ is true. We considered a range of values for c, with π={0.4,0.5,0.6} for the randomized study simulations, and for the observational study simulations propensity score models that, marginally, yield similar probabilities. We used synthetic regression forests from the R package randomForest-SRC (Ishwaran and Kogalur, 2019) to estimate $C_{0}(X)$ and τ(X).

We present results under Model 1 with π=0.5 and n=1000, where Model 1 takes $C_{0}\left(X_{i}\right)=3.18+0.2X_{1i}+X_{2i}+0.5X_{3i}$, and $\tau \left(X_{i}\right)=c1\left(-0.5X_{2i}^{2}+X_{4i}>0\right)$. We generated $X_{1},$ $X_{2},$ $X_{3}$ as independent N(0,1) and $X_{4},$ $X_{5}$ as independent Bernoulli with mean 0.5. Under Model 1 (scheme 1), about 58.9% of the population had a treatment effect of 0. Results under the other two models and settings are similar, and can be found in Web Tables 4– 15 of the Supporting Information.

As shown in Table 1, the type I error (α=0.05) under the randomized setting is controlled under all four versions of the test statistic. In the observational setting, it is controlled under all versions except for $T_{\text{IPW,}\;\text{SAP-match}}$ (see Web Table 2). The test statistics are slightly, negatively biased, which is to be expected when this one-step procedure is used in finite samples. The power using $T_{AIPW,•}$ is superior to that using $T_{\text{IPW,•}}$. Remarkably, under the randomized setting, $T_{\text{IPW,}\;\text{SAP-match}}$ has much higher power than $T_{\text{IPW,}\;\text{SBT}}$. This can be explained by Theorem 1. For $T_{\text{AIPW,•}}$, the power is similar across the SBT and SAP-match chunking methods. We empirically validated the result in Theorem 1 by running simulations that computed $T_{\text{IPW,}\;\text{SAP-match}}$ by using ${\hat{\sigma }}_{j}$ in place of ${\hat{\sigma }}_{j,m\pi }$. Refer to Web Table 1. We also considered a second data-generating scheme (scheme 2) for Model 1, where $X_{1}-X_{5}$ are as in scheme 1, and 20 additional iid *N*(0, 1) covariates were generated. As anticipated, the type I error remains controlled, and the power is almost the same as that under scheme 1. See Web Table 3 in the Supporting Information.

### 5.2 | Right-censored time-to-event

We examine the type I error rate and power of $T_{IPW,SBT}^{S},$ $T_{AIPW,SBT}^{S}$, and $T_{CAIPW,SBT}^{S}$. For brevity, we only ran simulations under the SBT chunking approach. For each simulation, data sets were generated according to the following accelerated failure time (AFT) model: $log\left(T_{i}\right)=1.75+0.5X_{1i}+X_{1i}^{2}+0.3X_{2i}+0.2X_{3i}+0.3X_{4i}+0.6X_{5i}+c*A_{i}*𝟙\left(X_{2}+3X_{4}>0\right)+e_{i}$, where c controls whether $H_{0}(c\leq 0)$ or $H_{1}(c>0)$ is true, and $X_{1}-X_{5}$ were generated as in Model 1 scheme 1 of Section 5.1. Under the above model, approximately 25% of the population had a treatment effect of exactly zero (based on 10,000 Monte Carlo samples). We considered two distributions for $e_{i}:e_{i}$ generated as N(0,0.25) (Model 1), or $e_{i}=log\left(Z_{i}\right)$, where $Z_{i}$ is generated as Exponential(1) (Model 2). Model 1 is not a proportional hazards model, whereas Model 2 is. We also varied the propensity model and the model for the censoring time. Specifically, $A_{i}$ is a Bernoulli random variable with mean $\pi \left(X_{i}\right)=0.5$ or $\pi \left(X_{i}\right)=exp(-0.3+\left.0.2X_{1i}+0.6X_{5i}\right)/\left\{1+exp\left(-0.3+0.2X_{1i}+0.6X_{5i}\right)\right\}$. The latter model, used to emulate observational data, is such that the marginal probability that A=1 is approximately 0.5. We considered three models for the censoring time $C_{i}$: (a) U(0,50), (b) U(0,100), and (c) exponential with rate $exp\left\{-1\left(3.5+0.1X_{1i}+0.2A_{i}\right)\right\}$. We use the notation “Model (1/2) (a/b/c) “ to refer to the combination of failure time model and censoring time model that is of interest. The cutoff time for computing RMST was chosen such that approximately 80–90% of the data are observed by time L. Censoring models (a) and (c) induce higher levels of censoring (by time L), compared with censoring model (b) (See Web Table 16).

We chose n, $l_{n},$ m, α, and the number of Monte Carlo data sets per simulation as in Section 5.1. The perturbation term, $\zeta_{i}$, used to compute $T_{AIPW,SBT}^{S}$ and $T_{CAIPW,SBT}^{S}$, was generated as Exponential(1). Random survival forest estimates were computed using the R package random-ForestSRC (Ishwaran and Kogalur, 2019). We present results under the observational data setting for Model 1 b and Model 1c. Results from the other models are similar and can be found in the Supporting Information. The type I error is controlled for all simulation scenarios and test statistics considered (see Tables 2, 3, and Web Tables 17– 27). Within each scenario, $T_{\text{CAIPW}}$ demonstrates more power to detect the alternative than $T_{IPW}$ and $T_{AIPW}$, and $T_{\text{IPW}}$ and $T_{\text{AIPW}}$ tend to yield identical power. Thus, it appears that any potential efficiency gains that may result from using $T_{\text{AIPW}}$ instead of $T_{\text{IPW}}$ are negated by the random perturbation term in the specification of $T_{AIPW}$. By comparing Table 2 with Table 3 (Web Tables 19, 22, and 26 with Web Tables 20, 23, and 27), one can see that the power gain from using $T_{CAIPW}$ instead of either $T_{IPW}$ or $T_{\text{AIPW}}$ becomes more pronounced as the amount of censoring increases. By comparing Model 1a with Model 1b (Web Tables 18 and 24 with Web Table 19 and Table 2) and Model 2a versus 2b (Web Tables 21 and 25 with Web Tables 22 and 26), one can see that as the percentage of censoring increases, the power of the test decreases. Finally, as in the uncensored outcome setting, the proposed test remains valid even as p increases from 5 to 25 (see Web Table 17). In Web Appendix L, we examine in both uncensored and censored data scenarios how the choice of m may affect the power of the proposed test via simulations with m={2,5,10,20}. The results in Web Table 29 show that power is insensitive to the choice of m.

## 6 | APPLICATION TO A PHASE III TRIAL

In the Phase III trial referenced in Lipkovich et al. (2017), 599 patients with hematological malignancies were randomized to either experimental therapy plus best supporting care (active treatment, n=303) or best supporting care (control, n=296). The hazard ratio (treatment vs. control) for overall survival was borderline significant (hazard ratio = 0.85, one-sided p=0.0367). We use the proposed one-step value difference test to test whether at least some subgroup benefits from the active treatment. Specifically, we are testing whether at least some subgroup has improved restricted mean survival time under active treatment, compared to control. We considered the same 14 covariates described in Lipkovich et al. (2017); refer to Web Appendix J for a list of the covariates. Eight patients had unknown prognostic score for myelodysplastic syndromes risk assessment, which was one of the covariates collected. Those eight patients were excluded from the proposed test. We chose L=510 days such that about 90% of the $U_{i}$ are observed by L. This is similar to how we chose L in our simulations, as it prevents ${\hat{K}}_{c,j}\left(U_{i}^{L}|X_{i},A_{i}\right)$ from being too close to zero. We let $l_{n}=291$ and m=10, and we randomly allocated the observed data to each of the 31 chunks. Results based on $T_{IPW,SBT}^{S},$ $T_{AIPW,SBT}^{S}$, and $T_{\text{CAIPW,}\;\text{SBT}}^{S}$ are presented in Table 4. Using the CAIPW estimator, we find evidence (α=0.05) that at least some patients have a larger RMST under active treatment versus control; however, we do not find such evidence when using the IPW or AIPW estimator. This emphasizes the importance of using the CAIPW estimator, especially when the data are heavily censored. For these data, approximately 83% of the event times were censored. Given that there is evidence of treatment benefit for at least some patients (based on CAIPW estimator), the next step would be to identify the subgroup(s) and/or estimate an optimal treatment regime. While not the focus of this paper, this can be done under a variety of approaches. For example, Laber and Zhao (2015), Tao et al. (2018), and Sun and Wang (2021) propose various tree-based methods for estimating optimal treatment rules. Refer to Web Appendix K (and corresponding outputs in Web Figures 2 and 3 and Web Table 28) for an additional example, this time involving an uncensored endpoint.

## 7 | DISCUSSION

We have proposed a one-step value difference test for the existence of a subgroup with a beneficial treatment effect that yields root-n-rate inference for $\Psi \left(d^{\text{opt}}\right)=V\left(d^{\text{opt}}\right)-V(0)$. The test is applicable to cases when the outcome of interest is an uncensored, continuous variable or when the outcome of interest is a right-censored time-to-event. While our implementation uses random forests to estimate $C_{0}(X)$ and τ(X), provided (C4) and (C5) of LL16 are met, alternative regression estimation techniques can be used. One of the benefits to using random forests is that they are more robust to the “curse of dimensionality” than some other nonparametric estimation methods. Extension of the proposed framework to accommodate more than two treatments may be feasible. For example, if two active treatments (“active 1” and “active 2”) are to be compared to a control, one may consider redefining the test statistic to be the arg max of two value difference test statistics—one based on a value difference estimator for active 1 versus control and the other based on a value difference estimator for active 2 versus control. Furthermore, building off the ideas in Zhang et al. (2013) for the uncensored outcomes setting, and Hager et al. (2018) and Jiang et al. (2017) for the censored outcome setting, one may consider extending the proposed subgroup test to multiple treatment stages. These are areas of future work.

## Supplementary Material

supplement

code

## Figures and Tables

**TABLE 1:** Randomized study results under Model 1 scheme 1, with π=0.5,α=0.05,n=1000

c	Method	$\mu_{\text{IPW}}$	$\mu_{\text{AIPW}}$	$\sigma_{\text{IPW}}$	$\sigma_{\text{AIPW}}$	$1\;-\;\beta_{\text{IPW}}/\alpha_{\text{IPW}}$	$1\;-\;\beta_{\text{AIPW}}/\alpha_{\text{AIPW}}$
0.30	SAP-match	1.61	2.50	1.01	1.01	0.51	0.80
0.30	SBT	0.62	2.55	1.00	0.99	0.15	0.81
0.40	SAP-match	2.29	3.39	1.01	1.00	0.73	0.96
0.40	SBT	0.92	3.44	1.00	1.01	0.21	0.97
0.50	SAP-match	2.92	4.40	0.94	1.02	0.92	1.00
0.50	SBT	1.17	4.43	1.00	1.01	0.30	1.00
0.00	SAP-match	0.03	−0.03	1.00	0.99	0.04	0.05
0.00	SBT	−0.03	0.02	1.01	1.00	0.06	0.06
−1.00	SAP-match	0.02	−0.06	0.99	1.00	0.04	0.05
−1.00	SBT	−0.05	−0.07	1.02	1.04	0.06	0.05
−2.00	SAP-match	0.03	−0.03	0.98	0.99	0.05	0.05
−2.00	SBT	−0.04	−0.00	1.02	1.03	0.06	0.06

*Note*:Mean (μ), standard deviation (σ), and power or type I error (1−β/α) of the one-step value difference test statistic based on 500 simulated data sets. Subscripts “IPW” and “AIPW” correspond to $T_{\text{IPW},•}$ and $T_{\text{AIPW},•}$, respectively. “Method” refers to chunking method. Largest standard error for μ, σ, and 1−β/α is 0.05, 0.07, and 0.02, respectively.

**TABLE 2:** Results under Model 1b, with $\pi \left(X_{i}\right)=exp\left(-0.3+0.2X_{1i}+0.6X_{5i}\right)/\left\{1+exp\left(-0.3+0.2X_{1i}+0.6X_{5i}\right)\right\}$ and *L* = 42

c	n	$\mu_{\text{IPW}}$	$\mu_{\text{AIPW}}$	$\mu_{\text{CAIPW}}$	$\sigma_{\text{IPW}}$	$\sigma_{\text{AIPW}}$	$\sigma_{\text{CAIPW}}$	$1\;-\;\beta_{\text{IPW}}/\alpha_{\text{IPW}}$	$1\;-\;\beta_{\text{AIPW}}/\alpha_{\text{AIPW}}$	$1\;-\;\beta_{\text{CAIPW}}/\alpha_{\text{CAIPW}}$
1.25	600	2.82	3.08	5.79	1.08	1.06	1.09	0.86	0.91	1.00
1.25	1000	3.82	4.03	7.56	1.03	1.02	1.14	0.98	0.99	1.00
0.75	600	1.85	2.09	3.71	1.08	1.06	1.05	0.57	0.67	0.97
0.75	1000	2.50	2.73	4.92	1.03	1.05	1.08	0.82	0.86	1.00
0.50	600	1.18	1.38	2.42	1.08	1.06	1.04	0.33	0.42	0.77
0.50	1000	1.68	1.87	3.27	1.06	1.05	1.04	0.53	0.58	0.95
0.00	600	−0.18	−0.03	−0.09	1.10	1.06	1.00	0.06	0.05	0.03
0.00	1000	−0.15	−0.03	−0.04	1.08	1.03	0.98	0.05	0.06	0.05
−0.50	600	−0.40	−0.22	−0.27	1.05	1.03	1.01	0.03	0.04	0.03
−0.50	1000	−0.33	−0.14	−0.19	1.03	0.97	0.98	0.03	0.03	0.03
−1.00	600	−0.32	−0.13	−0.14	1.07	1.10	1.07	0.02	0.06	0.05
−1.00	1000	−0.21	0.05	0.04	1.01	0.99	0.98	0.02	0.05	0.05

*Note*: Mean (μ), standard deviation (σ), and power or type I error (1−β/α) of the one-step value difference test statistic is based on 500 simulated data sets of *n* = {600, 1000}. Subscripts IPW, AIPW, and CAIPW specify whether the results are based on $T_{\text{IPW,SBT}}^{S}$,$T_{\text{AIPW, SBT}}^{S}$, or $T_{\text{CAIPW, SBT}}^{S}$, respectively. Largest standard error for μ and σ, and 1−β/α is 0.05 and 0.08, and 0.02, respectively.

**TABLE 3:** Results under Model 1c, with $\pi \left(X_{i}\right)=exp\left(-0.3+0.2X_{1i}+0.6X_{5i}\right)/\left\{1+exp\left(-0.3+0.2X_{1i}+0.6X_{5i}\right)\right\}$ and L=33

c	n	$\mu_{\text{IPW}}$	$\mu_{\text{AIPW}}$	$\mu_{\text{CAIPW}}$	$\sigma_{\text{IPW}}$	$\sigma_{\text{AIPW}}$	$\sigma_{\text{CAIPW}}$	$1\;-\;\beta_{\text{IPW}}/\alpha_{\text{IPW}}$	$1\;-\;\beta_{\text{AIPW}}/\alpha_{\text{AIPW}}$	$1\;-\;\beta_{\text{CAIPW}}/\alpha_{\text{CAIPW}}$
1.25	600	1.84	1.99	4.86	1.05	1.07	1.10	0.58	0.63	1.00
1.25	1000	2.62	2.72	6.48	1.08	1.06	1.12	0.81	0.86	1.00
0.75	600	1.24	1.39	3.24	1.06	1.07	1.03	0.35	0.39	0.93
0.75	1000	1.84	1.96	4.40	1.08	1.07	1.05	0.57	0.64	1.00
0.50	600	0.78	0.94	2.20	1.09	1.10	1.02	0.23	0.27	0.70
0.50	1000	1.27	1.38	2.97	1.08	1.08	1.04	0.37	0.40	0.89
0.00	600	−0.21	−0.08	−0.04	1.08	1.11	1.01	0.05	0.06	0.05
0.00	1000	−0.09	0.02	−0.02	1.08	1.09	1.06	0.05	0.06	0.06
−0.50	600	−0.42	−0.29	−0.31	1.06	1.08	1.07	0.03	0.04	0.03
−0.50	1000	−0.28	−0.10	−0.17	1.00	1.00	0.99	0.02	0.04	0.03
−1.00	600	−0.30	−0.15	−0.17	1.08	1.10	1.06	0.04	0.05	0.05
−1.00	1000	−0.19	0.04	0.03	1.03	0.98	0.99	0.02	0.06	0.05

*Note*: Entries are as in Table 2.

**TABLE 4:** Results from testing the hypothesis in (2) on the Phase III trial data

Estimator	Test statistic	*p*-Value
IPW	−2.91	0.998
AIPW	−2.94	0.998
CAIPW	1.66	0.0481

## Data Availability

The data that support the findings in this paper are available as follows. The data analyzed in Section 6 are available by request from the authors of Lipkovich et al. (2017). The data from AIDS Clinical Trials Group 175 analyzed in Web Appendix K are available on request from the AIDS Clinical Trials Group (https://actgnetwork.org) and from the National Technical Information Service (https://ntis.gov).
