# Supplementary material for: A general framework for subgroup detection via one-step value difference estimation
Source: Biometrics. Author manuscript; Available in PMC 2023 Dec 4. (PMC10694635; doi:10.1111/biom.13711)
Supplement: supplement [file NIHMS1945184-supplement-supplement.pdf]

BIOMETRICS 000, 000–000 DOI: 000
000 0000

Supporting Information for A General Framework for Subgroup Detection via One-step Value Difference Estimation by Dana Johnson, Wenbin Lu, and Marie Davidian

This paper has been submitted for consideration for publication in *Biometrics*

Web Appendix A: Proof that $\tau(\mathbf{X})$ Can be Identified in Terms of the Observed Data when the Outcome of Interest is a Right-censored Time-to-event

Under noninformative censoring, the cause-specific hazard for failing,

$$\lambda(t, \delta \mid \mathbf{X}, A) = \lim_{h \to 0} \frac{\mathrm{pr}(t \leqslant U < t + h, \Delta = \delta \mid U \geqslant t, \mathbf{X}, A)}{h},$$

with $\delta = 1$, is equal to the underlying hazard of failing defined as:

$$\lambda(t \mid \mathbf{X}, A) = \lim_{h \to 0} \frac{\mathrm{pr}(t \leqslant T < t + h \mid T \geqslant t, \mathbf{X}, A)}{h}.$$

This implies that

$$\Lambda(t, 1 \mid \mathbf{X}, A) = \Lambda(t \mid \mathbf{X}, A) = \int_0^t \lambda(z \mid \mathbf{X}, A) dz.$$

We can then show

$$\begin{aligned}
E\Big[\min\{T^*(1), L\} \mid \mathbf{X}\Big] &= E\Big[\min\{T^*(1), L\} \mid \mathbf{X}, A = 1\Big] \text{ by No Unmeasured Confounders} \\
&= E\Big[\min\{T, L\} \mid \mathbf{X}, A = 1\Big] \text{ by Consistency} \\
&= \int_0^L S(t \mid \mathbf{X}, A = 1) dt \\
&= \int_0^L \exp\{-\Lambda(t \mid \mathbf{X}, A = 1)\} dt \\
&= \int_0^L \exp\{-\Lambda(t, 1 \mid \mathbf{X}, A = 1)\} dt \text{ by Noninformative Censoring}
\end{aligned}$$

A similar argument can be made to show that $E\Big[\min\{T^*(0), L\} \mid \mathbf{X}\Big] = \int_0^L \exp\{-\Lambda(t, 1 \mid \mathbf{X}, A = 0)\} dt$. Therefore,

$$\begin{aligned}
\tau(\mathbf{X}) &= E\Big[\min\{T^*(1), L\} \mid \mathbf{X}\Big] - E\Big[\min\{T^*(0), L\} \mid \mathbf{X}\Big] \\
&= \int_0^L \exp\{-\Lambda(t, 1 \mid \mathbf{X}, A = 1)\} dt - \int_0^L \exp\{-\Lambda(t, 1 \mid \mathbf{X}, A = 0)\} dt.
\end{aligned}$$

Web Appendix B: The Need for the Perturbation Term, ζ, When Extending the Framework to Survival Data

Censoring adds an extra layer of difficulty when trying to derive a regular estimator for the value difference under the optimal rule. We handle this difficulty by including a perturbation

term in the AIPW and CAIPW versions of the value difference estimator for the case when the endpoint of interest is a right-censored time-to-event.

Consider the setting where the endpoint of interest is some right-censored time-to-event. Recall that for $j = 1, \ldots, r_n$, $\tau(\mathbf{X})$ is estimated by first building a random survival forest estimator for $S(t \mid \mathbf{X}, A = 0)$ using only the observations in O^*_{j-1} such that $A = 0$, and building a random survival forest estimator for $S(t \mid \mathbf{X}, A = 1)$ using only the observations in O^*_{j-1} such that $A = 1$. These estimators are denoted by $\widehat{S}_j(t \mid \mathbf{X}, A = 0)$ and $\widehat{S}_j(t \mid \mathbf{X}, A = 1)$, respectively. Then, we estimate $\tau(\mathbf{X})$ with $\widehat{\tau}_j(\mathbf{X}) = \int_0^L \widehat{S}_j(t \mid \mathbf{X}, A = 1) - \int_0^L \widehat{S}_j(t \mid \mathbf{X}, A = 0)$.

Assume the null hypothesis is true, where specifically, we assume there are some patients with $\mathbf{x} \in \mathcal{X} : \tau(\mathbf{x}) < 0$ and some patients with $\mathbf{x} \in \mathcal{X} : \tau(\mathbf{x}) = 0$. Furthermore, consider the extreme case where all events corresponding to patients with $\mathbf{x} \in \mathcal{X} : \tau(\mathbf{x}) = 0$ are censored. Then the random survival forest built to estimate $S(t \mid \mathbf{X}, A = 1)$ will rely heavily on the information from patients with $\mathbf{x} \in \mathcal{X} : \tau(\mathbf{x}) < 0$. This means it is very likely that patients with $\mathbf{x} : \tau(\mathbf{x}) = 0$ will have have $\widehat{\tau}_j(\mathbf{x}) < 0$. When $1\{\widehat{\tau}_j(\mathbf{x}) > 0\} = 0$ for all observations in a chunk, $\widehat{\Psi}_{j,\bullet}(\mathcal{C}_j, \mathcal{O}^*_{j-1})$ is exactly zero, which disrupts the behavior of the estimator (cannot necessarily apply distributional result to the finite sample), and this is likely to happen under the setting just described. Even if we relax the assumption that *all* events corresponding to patients with $\mathbf{x} \in \mathcal{X} : \tau(\mathbf{x}) = 0$ are censored, the issues described above may persist. In fact, we encountered these issues in our own simulations, before we realized that we needed to include a perturbation term in the AIPW and CAIPW versions of our value difference estimator.

Web Appendix C: Estimating $C_0(\mathbf{X})$ and $\tau(\mathbf{X})$ Using the Counterfactual Synthetic Random Forest Approach

When the outcome of interest is uncensored and real-valued, we use the counterfactual *synthetic* random forest approach described in Lu *et al.* (2018), instead of the counterfactual random forest approach (used for time-to-event data), as it generally performed better in terms of bias and root mean squared error compared to other random forest methods examined in that paper– including virtual twins (Foster *et al.*, 2011), bivariate imputation random forests (Lu *et al.*, 2018), honest random forests (Wager and Athey, 2018), and bayesian adaptive regression trees (Hill, 2011). To clarify, the counterfactual synthetic random forest approach differs from the standard counterfactual random forest approach in that the regression forests used in the former approach are *synthetic* regression forests instead of traditional regression forests. Synthetic random forests can be thought of as a hyperforest, in that they are built using a collection of Breiman random forests that are grown under different values of tuning parameters, such as "nodesize" and "mtry". For example, the synthetic regression forests used in this paper were built under the following values for the nodesize parameter: 1-10, 20, 30, 50, 100. After growing these forests, which each produce their own predicted values, the final synthetic forest is grown by including as covariates both $\mathbf{X}$ *and* the predicted values from the forests generated under the varying tuning parameter values. Synthetic forests can be constructed using the R (R Core Team, 2020) package randomForestSRC (Ishwaran and Kogalur, 2007; Ishwaran *et al.*, 2008; Ishwaran and Kogalur, 2019). To our knowledge, an implementation of synthetic forests for survival data are not yet available, which is why we only consider this approach for uncensored scalar outcomes.

Web Appendix D: Estimating $C_0(\mathbf{X})$ and $\tau(\mathbf{X})$ Using the Counterfactual Random Survival Forest Approach

To our knowledge, software to construct synthetic random forests is available only for the regular regression setting but not for survival data. For this reason, when dealing with survival data we use the counterfactual (instead of synthetic counterfactual) random forest approach described by Lu *et al.* (2018) to estimate $C_0(\mathbf{X})$ and $\tau(\mathbf{X})$. Specifically, the observations with $A = a, a = 0, 1$, are used separately to build random survival forests that estimate the conditional survival distribution; denote these as $\widehat{S}(t \mid \mathbf{X}, a), a = 0, 1$. The estimator for $C_0(\mathbf{X})$ is then $\widehat{C}_0(\mathbf{X}) = \int_0^L \widehat{S}(t \mid \mathbf{X}, A = 0)dt$, and the estimator for the CATE is $\widehat{\tau}(\mathbf{X}) = \int_0^L \widehat{S}(t \mid \mathbf{X}, A = 1)dt - \int_0^L \widehat{S}(t \mid \mathbf{X}, A = 0)dt$.

Web Appendix E: Decomposition of the Censoring Augmentation Term

The censoring augmentation term (the part that is an integral), which is a function of nuisance functions, can be rewritten as:

$$\int_0^L \frac{\mathrm{d}\widehat{M}_{c,j}(r \mid \mathbf{X}_i, A_i)}{\widehat{K}_{c,j}(r \mid \mathbf{X}_i, A_i)} \widehat{m}_j(r \mid \mathbf{X}_i, A_i) = \frac{(1 - \Delta_i^L)\widehat{m}_j(U_i^L \mid \mathbf{X}_i, A_i)}{\widehat{K}_{c,j}(U_i^L \mid \mathbf{X}_i, A_i)} - \int_0^{U_i^L} \frac{d\widehat{\Lambda}_{c,j}(r \mid \mathbf{X}_i, A_i)}{\widehat{K}_{c,j}(r \mid \mathbf{X}_i, A_i)} \widehat{m}_j(r \mid \mathbf{X}_i, A_i). \quad (1)$$

Proof

Recall $\mathrm{d}M_c(r \mid \mathbf{X}_i, A_i) = \mathrm{d}N_c(r) - \lambda_c(r \mid \mathbf{X}_i, A_i)Y(r)\mathrm{d}r$, $N_c(r) = 1(U_i \leqslant r, \Delta_i = 0)$ is the censoring counting process, $Y(r) = 1(U_i \geqslant r)$ is the at risk process, and $m(r \mid \mathbf{X}_i, A_i) = E\{\min(T, L) \mid T \geqslant r, \mathbf{X}_i, A_i\}$. Consider the following quantity:

$$\int_0^L \frac{\mathrm{d}N_c(r)}{K_c(r \mid \mathbf{X}_i, A_i)}. \quad (2)$$

The above will be nonzero only if patient i has $\Delta_i = 0$ and $U_i \leqslant L$ (since the integral only goes up to L). It turns out that the only time $\Delta_i = 0$ and $U_i \leqslant L$ is when $\Delta_i^L = 0$. Thus,

(2) can be rewritten as:

$$\frac{1-\Delta_i^L}{K_c(U_i \mid \mathbf{X}_i, A_i)} = \frac{1-\Delta_i^L}{K_c(U^L \mid \mathbf{X}_i, A_i)},$$

since $U_i = U_i^L$ when $U_i \leqslant L$.

For patient i:

$$\int_0^L \frac{\mathrm{d}\widehat{M}_{c,j}(r \mid \mathbf{X}_i, A_i)}{\widehat{K}_{c,j}(r \mid \mathbf{X}_i, A_i)} \widehat{m}_j(r \mid \mathbf{X}_i, A_i) =$$

$$\int_0^L \frac{\{\mathrm{d}N_c(r) - \widehat{\lambda}_{c,j}(r \mid \mathbf{X}_i, A_i) Y(r)\mathrm{d}r\}}{\widehat{K}_{c,j}(r \mid \mathbf{X}_i, A_i)} \widehat{m}_j(r \mid \mathbf{X}_i, A_i)$$

$$= \int_0^L \frac{\mathrm{d}N_c(r)}{\widehat{K}_{c,j}(r \mid \mathbf{X}_i, A_i)} \widehat{m}_j(r \mid \mathbf{X}_i, A_i) - \int_0^L \frac{\widehat{\lambda}_{c,j}(r \mid \mathbf{X}_i, A_i) Y(r)\mathrm{d}r}{\widehat{K}_{c,j}(r \mid \mathbf{X}_i, A_i)} \widehat{m}_j(r \mid \mathbf{X}_i, A_i)$$

$$= \frac{(1-\Delta_i^L)\widehat{m}_j(U_i^L \mid \mathbf{X}_i, A_i)}{\widehat{K}_{c,j}(U_i^L \mid \mathbf{X}_i, A_i)} - \int_0^{U_i^L} \frac{\widehat{\lambda}_{c,j}(r \mid \mathbf{X}_i, A_i)\mathrm{d}r}{\widehat{K}_{c,j}(r \mid \mathbf{X}_i, A_i)} \widehat{m}_j(r \mid \mathbf{X}_i, A_i)$$

$$= \frac{(1-\Delta_i^L)\widehat{m}_j(U_i^L \mid \mathbf{X}_i, A_i)}{\widehat{K}_{c,j}(U_i^L \mid \mathbf{X}_i, A_i)} - \int_0^{U_i^L} \frac{d\widehat{\Lambda}_{c,j}(r \mid \mathbf{X}_i, A_i)}{\widehat{K}_{c,j}(r \mid \mathbf{X}_i, A_i)} \widehat{m}_j(r \mid \mathbf{X}_i, A_i).$$

Web Appendix F: One-Step Framework for Uncensored Data

Recall we first estimate the nuisance functions: $\pi(\mathbf{X})$, $\pi_A(\mathbf{X})$, $\tau(\mathbf{X})$, and $C_0(\mathbf{X})$ using a 'chunk' of l_n observations. We partition the remaining data into $r_n = (n - l_n)/m$ chunks of size m, where $m \geqslant 1$, and l_n is chosen such that $(n - l_n)/m \to \infty$ as $n \to \infty$. Let $\mathcal{C}_j = \{O_i : O_i \in j\text{th data chunk}\}$, so that each $\mathcal{C}_j$ for $j = 1, \ldots, r_n$ contains m observations. We also let $\mathcal{O}_j^* = \left\{O_i : O_i \in \bigcup_{k=0}^{j} \mathcal{C}_k\right\}$, where $\mathcal{C}_0 \equiv \mathcal{O}_0^*$ corresponds to all observations in the initial data chunk of size l_n. Define

$$S_{j,\mathrm{IPW}}(O_i, \mathcal{O}_{j-1}^*) = \left\{ \frac{1[A_i = 1\{\widehat{\tau}_j(\mathbf{X}_i) > 0\}]}{\widehat{\pi}_{j,A_i}(\mathbf{X}_i)} Y_i - \left(\frac{1[A_i = 1\{\widehat{\tau}_j(\mathbf{X}_i) > 0\}]}{\widehat{\pi}_{j,A_i}(\mathbf{X}_i)} - 1 \right) \right.$$
$$\left. \times \left[\widehat{C}_{j,0}(\mathbf{X}_i) + \widehat{\tau}_j(\mathbf{X}_i) 1\{\widehat{\tau}_j(\mathbf{X}_i) > 0\} \right] \right\} - \left\{ \frac{1(A_i = 0)}{1 - \widehat{\pi}_j(\mathbf{X}_i)} Y_i \right\},$$

$$S_{j,\mathrm{AIPW}}(O_i,\mathcal{O}^*_{j-1}) = \Bigg\{\frac{1[A_i = 1\{\widehat{\tau}_j(\mathbf{X}_i)>0\}]}{\widehat{\pi}_{j,A_i}(\mathbf{X}_i)}Y_i - \Big(\frac{1[A_i = 1\{\widehat{\tau}_j(\mathbf{X}_i)>0\}]}{\widehat{\pi}_{j,A_i}(\mathbf{X}_i)} - 1\Big)$$

$$\times\Big[\widehat{C}_{j,0}(\mathbf{X}_i) + \widehat{\tau}_j(\mathbf{X}_i)1\{\widehat{\tau}_j(\mathbf{X}_i)>0\}\Big]\Bigg\} - \Bigg[\frac{1(A_i=0)}{1-\widehat{\pi}_j(\mathbf{X}_i)}Y_i - \Big\{\frac{1(A_i=0)}{1-\widehat{\pi}_j(\mathbf{X}_i)} - 1\Big\}\widehat{C}_{j,0}(\mathbf{X}_i)\Bigg],$$

and

$$\widehat{\Psi}_{j,\bullet}(\mathcal{C}_j,\mathcal{O}^*_{j-1}) = m^{-1}\sum_{\mathcal{C}_j} S_{j,\bullet}(O_i,\mathcal{O}^*_{j-1}),$$

where $\widehat{\pi}_j(\mathbf{X}), \widehat{\pi}_{j,A}(\mathbf{X}), \widehat{\tau}_j(\mathbf{X})$, and $\widehat{C}_{j,0}(\mathbf{X})$ are estimators for $\pi(\mathbf{X}), \pi_A(\mathbf{X}), \tau(\mathbf{X})$, and $C_0(\mathbf{X})$, respectively, based on the 'historical' data in $\mathcal{O}^*_{j-1}$. At step j, these estimators are known, fixed functions.

Then the SBT version of our test statistic is:

$$T_{\bullet,\mathrm{SBT}} = r_n^{-1/2}\sum_{j=1}^{r_n}\widehat{\sigma}_j^{-1}\widehat{\Psi}_{j,\bullet}(\mathcal{C}_j,\mathcal{O}^*_{j-1}) = r_n^{-1/2}\Big(\sum_{j=1}^{r_n}\widehat{\sigma}_j^{-1}\Big)\widehat{\Psi}(O), \tag{3}$$

$$\widehat{\Psi}(O) = \frac{\sum_{j=1}^{r_n}\widehat{\sigma}_j^{-1}\widehat{\Psi}_{j,\bullet}(\mathcal{C}_j,\mathcal{O}^*_{j-1})}{\sum_{j=1}^{r_n}\widehat{\sigma}_j^{-1}},$$

and the SAP-match version of our test statistic is:

$$T_{\bullet,\text{SAP-match}} = r_n^{-1/2}\sum_{j=1}^{r_n}(\widehat{\sigma}_j^{m\pi})^{-1}\widehat{\Psi}^{m\pi}_{j,\bullet}(\mathcal{C}_j,\mathcal{O}^*_{j-1}) = r_n^{-1/2}\Big\{\sum_{j=1}^{r_n}(\widehat{\sigma}_j^{m\pi})^{-1}\Big\}\widehat{\Psi}^{m\pi}(O), \tag{4}$$

$$\widehat{\Psi}^{m\pi}(O) = \frac{\sum_{j=1}^{r_n}(\widehat{\sigma}_j^{m\pi})^{-1}\widehat{\Psi}^{m\pi}_{j,\bullet}(\mathcal{C}_j,\mathcal{O}^*_{j-1})}{\sum_{j=1}^{r_n}(\widehat{\sigma}_j^{m\pi})^{-1}}.$$

Web Appendix G: Conditions Under Which Random Forests May Satisfy (C4) and (C5) of Luedtke and van der Laan (2016)

Wager and Athey (2018) found that, under certain constraints on the subsampling rate s_n, which is the number of observations subsampled from the observed data and used to build a single tree, random forest predictions converge at the rate $n^{\beta-1/2}$, where β is chosen to satisfy (14) in their paper. We assume that, under this rate, conditions (C4) and (C5) of

Luedtke and van der Laan (2016) are satisfied, namely that both R_{1n} and R_{2n} in their paper are $o_p(n^{-1/2})$. Our simulations in Section 5.1 suggest this assumption holds.

Web Appendix H: Proof that $E\Big\{\widehat{\Psi}^{S,m\pi}_{j,\bullet}(\mathcal{C}_j, \mathcal{O}^*_{j-1}) \mid \mathcal{O}^*_{j-1}\Big\} = E\Big\{\widehat{\Psi}^{S}_{j,\bullet}(\mathcal{C}_j, \mathcal{O}^*_{j-1}) \mid \mathcal{O}^*_{j-1}\Big\}$

Proof

$$
\begin{aligned}
E\Big\{\hat{\Psi}^{S,m\pi}_{j,\bullet}(\mathcal{C}_j, \mathcal{O}^*_{j-1}) \mid \mathcal{O}^*_{j-1}\Big\} &= E\Bigg[m^{-1}\Big\{\sum_{\mathcal{C}_j} 1(A_i = 1) S^S_{j,\bullet}(O_i, \mathcal{O}^*_{j-1}) \\
&\quad + \sum_{\mathcal{C}_j} 1(A_i = 0) S^S_{j,\bullet}(O_i, \mathcal{O}^*_{j-1})\Big\}\Big| \mathcal{O}^*_{j-1}, \sum_{\mathcal{C}_j} A_i = m\pi\Bigg] \\
&= m^{-1}\Bigg[m\pi E\Big\{S^S_{j,\bullet}(O_i, \mathcal{O}^*_{j-1}) \mid \mathcal{O}^*_{j-1}, A_i = 1\Big\} + m(1-\pi) \\
&\quad \times E\Big\{S^S_{j,\bullet}(O_i, \mathcal{O}^*_{j-1}) \mid \mathcal{O}^*_{j-1}, A_i = 0\Big\}\Bigg] \\
&= \pi E\Big\{S^S_{j,\bullet}(O_i, \mathcal{O}^*_{j-1}) \mid \mathcal{O}^*_{j-1}, A_i = 1\Big\} + (1-\pi) \\
&\quad \times E\Big\{S^S_{j,\bullet}(O_i, \mathcal{O}^*_{j-1}) \mid \mathcal{O}^*_{j-1}, A_i = 0\Big\} \\
&= \mathrm{pr}(A_i = 1 \mid \mathcal{O}^*_{j-1}) E\Big\{S^S_{j,\bullet}(O_i, \mathcal{O}^*_{j-1}) \mid \mathcal{O}^*_{j-1}, A_i = 1\Big\} \\
&\quad + \mathrm{pr}(A_i = 0 \mid \mathcal{O}^*_{j-1}) E\Big\{S^S_{j,\bullet}(O_i, \mathcal{O}^*_{j-1}) \mid \mathcal{O}^*_{j-1}, A_i = 0\Big\} \\
&\quad \text{, since } A_i \perp\!\!\!\perp \mathcal{O}^*_{j-1} \\
&= E\Big\{S^S_{j,\bullet}(O_i, \mathcal{O}^*_{j-1}) \mid \mathcal{O}^*_{j-1}\Big\} \text{ by the Law of Total Expectation} \\
&= E\Big\{\hat{\Psi}^S_{j,\bullet}(\mathcal{C}_j, \mathcal{O}^*_{j-1}) \mid \mathcal{O}^*_{j-1}\Big\}.
\end{aligned}
$$

Web Appendix I: Proof of Theorem 1

Conditions for Theorem 1:

- Stable unit treatment value/ consistency assumption (Rubin, 1974; Cole and Hernán, 2008)
  - For uncensored outcome: $Y = Y^*(A)$

– For time-to-event: $T = T^*(1)1(A=1) + T^*(0)1(A=0)$, and $C = C^*(1)1(A=1) + C^*(0)1(A=0)$

- Positivity assumption (Hernán and Robins, 2006)

  – $\text{pr}(A = a \mid \mathbf{X} = \mathbf{x}) > 0$ for all $\mathbf{x} \in \mathcal{X}$

- No unmeasured confounders assumption (Cole and Hernán, 2008)

  – For uncensored outcome: $\{Y^*(0), Y^*(1)\} \perp\!\!\!\perp A \mid \mathbf{X}$, where $\perp\!\!\!\perp$ means "independent" and the notation $\cdot \perp\!\!\!\perp \cdot \mid \mathbf{X}$ refers to conditional independence given $\mathbf{X}$.

  – For time-to-event: $\{T^*(0), T^*(1)\} \perp\!\!\!\perp A|\mathbf{X}$

- Noninformative censoring (for time-to-event data only)

  – $C \perp\!\!\!\perp \{T^*(0), T^*(1)\} \mid \mathbf{X}$

THEOREM 1: $\text{var}\Big\{\widehat{\Psi}^{S}_{j,\bullet}(\mathcal{C}_j, \mathcal{O}^*_{j-1}) \mid \mathcal{O}^*_{j-1}\Big\} \geqslant \text{var}\Big\{\widehat{\Psi}^{S,m\pi}_{j,\bullet}(\mathcal{C}_j, \mathcal{O}^*_{j-1}) \mid \mathcal{O}^*_{j-1}\Big\}$

Proof

$$\begin{aligned}
\text{var}\Big\{\widehat{\Psi}^{S}_{j,\bullet}(\mathcal{C}_j, \mathcal{O}^*_{j-1}) \mid \mathcal{O}^*_{j-1}\Big\} &= m^{-1}\text{var}\Big\{S^{S}_{j,\bullet}(O_i, \mathcal{O}^*_{j-1}) \mid \mathcal{O}^*_{j-1}\Big\} \\
&= m^{-1}\Bigg[\pi\text{var}\Big\{S^{S}_{j,\bullet}(O_i, \mathcal{O}^*_{j-1}) \mid \mathcal{O}^*_{j-1}, A_i = 1\Big\} + (1-\pi)\text{var}\Big\{S^{S}_{j,\bullet}(O_i, \mathcal{O}^*_{j-1}) \mid \mathcal{O}^*_{j-1}, A_i = 0\Big\}\Bigg] \\
&\quad + m^{-1}\text{var}\Bigg[E\Big\{S^{S}_{j,\bullet}(O_i, \mathcal{O}^*_{j-1}) \mid \mathcal{O}^*_{j-1}, A_i\Big\} \mid \mathcal{O}^*_{j-1}\Bigg] \text{ by Law of Total Variance} \\
&= \text{var}\Big\{\widehat{\Psi}^{S,m\pi}_{j,\bullet}(\mathcal{C}_j, \mathcal{O}^*_{j-1}) \mid \mathcal{O}^*_{j-1}\Big\} + m^{-1}\text{var}\Bigg[E\Big\{S^{S}_{j,\bullet}(O_i, \mathcal{O}^*_{j-1}) \mid \mathcal{O}^*_{j-1}, A_i\Big\} \mid \mathcal{O}^*_{j-1}\Bigg] \\
&\geqslant \text{var}\Big\{\widehat{\Psi}^{S,m\pi}_{j,\bullet}(\mathcal{C}_j, \mathcal{O}^*_{j-1}) \mid \mathcal{O}^*_{j-1}\Big\}.
\end{aligned}$$

Web Appendix J: Application to Phase III Clinical Trial (Censored Health Outcome)

We apply our proposed test to data from a Phase III clinical trial in patients with hematological malignancies (Lipkovich *et al.*, 2017). The covariate vector $\mathbf{X}_i$ contains the following

baseline information: sex (male, female), race (asian, black, white), cytogenetic category (very good, good, intermediate, poor, very poor), cytogenetic marker 1 (absent, present), cytogenetic marker 2 (absent, present), cytogenetic marker 3 (absent, present), cytogenetic marker 4 (absent, present), cytogenetic marker 5 (absent, present), cytogenetic marker 6 (absent, present), cytogenetic marker 7 (absent, present), cytogenetic marker 8 (absent, present), cytogenetic marker 9 (absent, present), prognostic score for myelodysplastic syndromes risk assessment (IPSS-R) (low, intermediate, high, very high), and outcome for patient's prior therapy (failure, progression, relapse). We let $l_n = 291$ and $m = 10$, and we randomly allocate the observed data to each of the 31 chunks (initial chunk plus 30 subsequent chunks).

Web Appendix K: Application to AIDS Clinical Trial (Uncensored Health Outcome)

We apply the proposed test (for uncensored endpoint) to data on 1046 patients from the AIDS Clinical Trials Group Protocol 175 (ACTG175). In this trial, subjects were randomly assigned, with equal probability, to one of four treatments: zidovudine monotherapy, zidovudine + didanosine, zidovudine + zalcitabine, or didanosine monotherapy. A large set of baseline information was collected on each patient, and the purpose of the study was to use the observed data to compare the effects of antiretroviral monototherapies with combination therapies in HIV-1-infected patients (Hammer *et al.*, 1996). Lu *et al.* (2013) considered the subset of patients who received either zidovudine + didanosine (active treatment, 522 patients) or zidovudine + zalcitabine (control, 524 patients) and estimated the optimal treatment regime using their loss-based variable selection method. They found evidence of a subgroup that benefits from treatment, where subgroup membership is a function of age and homosexual activity. We also consider this subset and apply our one-step value difference test.

Following Tsiatis *et al.* (2008), Zhang *et al.* (2008), and Lu *et al.* (2013), we consider CD4

count (cells/mm^3) at 20 $\pm$ 5 weeks to be the continuous response of interest and include 12 baseline covariates in the model, five continuous covariates, CD4 count (cells/mm^3) at baseline, CD8 count (cells/mm^3) at baseline, age (years), Karnofsky score (0–100), and weight (kg); and seven binary covariates, race (0 = white, 1 = non-white), gender (0 = female, 1 = male), hemophelia, homosexual activity, history of intravenous drug use, antiretroviral history (0 = naive, 1 = experienced), and symptomatic status.

We conduct the test under all four versions of the test statistic. For the SAP-match chunking approach, the initial chunk is composed of 272 patients from the zidovudine + didanosine group and 274 patients from the zidovudine + zalcitabine group, and there are 50 subsequent chunks, each of size 10, where half of the observations are treatment observations and half are control. This is done to maintain the randomization proportion used in the study, which is $\pi = 0.5$. For the SBT chunking approach, we use the same number of observations in the initial chunk and subsequent chunks as we did for method 2, but now we do not pay attention to treatment labels when building the chunks. The test statistics and associated p-values based on $T_{\text{IPW, SAP-match}}, T_{\text{IPW, SBT}}, T_{\text{AIPW, SAP-match}}$, and $T_{\text{AIPW, SBT}}$ are presented in Table 28, which also contains the results when the treatment labels are switched, i.e., zidovudine + zalcitabine is considered active treatment.

With zidovudine + didanosine as treatment and $T_{\text{IPW, SAP-match}}$ as the test statistic, we find strong evidence (p-value < 0.01) of a subgroup that benefits from treatment, but we do not find evidence of a subgroup when we use $T_{\text{IPW, SBT}}$ (p-value of 0.17). This is consistent with the striking power differences we observed in our simulation studies comparing $T_{\text{IPW, SAP-match}}$ against $T_{\text{IPW, SBT}}$, in the randomized setting. When we instead consider the results under $T_{\text{AIPW, SAP-match}}$ and $T_{\text{AIPW, SBT}}$, with zidovudine + didanosine as treatment, we find evidence of a subgroup that benefits from treatment regardless of how the data are allocated to chunks. This emphasizes the importance of using $T_{\text{AIPW},\bullet}$ instead of $T_{\text{IPW},\bullet}$, in practice.

After rejecting the null hypothesis, subgroup membership can be estimated by $1(\widehat{\tau}(\mathbf{X}) > 0)$, where $\widehat{\tau}(\mathbf{X})$ is the counterfactual random forest estimator for $\tau(\mathbf{X})$, computed using the full data. We estimate that 815 of the patients belong to the subgroup that benefits from treatment (zidovudine + didanosine) and that 231 should receive control (zidovudine + zalcitabine). To gain insight into which covariates are driving the treatment decision, we run a classification tree using the R (R Core Team, 2019) package rpart (Therneau and Atkinson, 2019). Figure 2 displays the treatment decision rule estimated by the tree. Age appears towards the top of the tree, which suggests it is an important variable for determining treatment allocation (Ishwaran *et al.*, 2010). The density plot in Figure 3 suggests that patients who should receive treatment are older, on average, than those who should receive control. This agrees with the optimal treatment rule estimated in Lu *et al.* (2013), which assigns younger patients to zidovudine + zalcitabine.

Finally, when the treatment labels are switched so that zidovudine + zalcitabine is the active treatment, we do not find evidence of a subgroup (refer to Table 28). This can be explained by insufficient power to detect such a small subgroup of patients that may benefit from zidovudine + zalcitabine.

Web Appendix L: Power as a function of m

Recall that in one-step estimation you execute the following steps repeatedly: 1.) estimate/re-estimate nuisance functions (initially just using a set of l_n observations) 2.) compute the chunk-specific value difference estimator, treating the estimated nuisance functions as fixed 3.) add a 'chunk' of data to the set of observations used to estimate the nuisance functions. If, for example, $n = 80$, this means that:

- If m = 2, the nuisance functions for the final chunk-specific value difference estimator will be based on a sample size of 80 - 2 = 78 observations

- If m = 5, the nuisance functions for the final chunk-specific value difference estimator will be based on a sample size of 80 - 5 = 75 observations
- If m = 10, the nuisance functions for the final chunk-specific value difference estimator will be based on a sample size of 80 -10 = 70 observations
- If m = 20, the nuisance functions for the final chunk-specific value difference estimator will be based on a sample size of 80 -20 = 60 observations

Essentially, m dictates how many observations will never enter into the 'pool of observations' used to estimate the nuisance functions. If l_n is large enough that nuisance functions are already being estimated well from the get-go, then leaving off 2 observations vs. 20 observations in the final step should not drastically affect your final test statistic and thus should not drastically affect power.

We explored how the choice of m may affect the power of the proposed test by running simulations with $m = \{2, 5, 10, 20\}$. At least in the settings we consider, the power is not affected by the choice of m. Please refer to Web Table 29.

[Table 1 about here.]

[Table 2 about here.]

[Table 3 about here.]

[Table 4 about here.]

[Table 5 about here.]

[Table 6 about here.]

[Table 7 about here.]

[Table 8 about here.]

[Table 9 about here.]

[Table 10 about here.]

[Table 11 about here.]

[Table 12 about here.]

[Table 13 about here.]

[Table 14 about here.]

[Table 15 about here.]

[Table 16 about here.]

[Table 17 about here.]

[Table 18 about here.]

[Table 19 about here.]

[Table 20 about here.]

[Table 21 about here.]

[Table 22 about here.]

[Table 23 about here.]

[Table 24 about here.]

[Table 25 about here.]

[Table 26 about here.]

[Table 27 about here.]

[Table 28 about here.]

[Figure 1 about here.]

[Figure 2 about here.]

[Figure 3 about here.]

References

Cole, S. R. and Hernán, M. A. (2008). Constructing inverse probability weights for marginal structural models. *American Journal of Epidemiology* **168,** 656–64.

Foster, J. C., Taylor, J. M. G., and Ruberg, S. J. (2011). Subgroup identification from randomized clinical trial data. *Statistics in Medicine* **30,** 2867–80.

Hammer, S. M., Katzenstein, D. A., Hughes, M. D., Gundacker, H., Schooley, R. T., Haubrich, R. H., Henry, W. K., Lederman, M. M., Phair, J. P., Niu, M., Hirsch, M. S., and Merigan, T. C. (1996). A trial comparing nucleoside monotherapy with combination therapy in HIV-infected adults with CD4 cell counts from 200 to 500 per cubic millimeter. *New Engl. J. Med.* **335,** 1081–90.

Hernán, M. A. and Robins, J. M. (2006). Estimating causal effects from epidemiological data. *J. Epidemiol. Commun. H.* **60,** 578–86.

Hill, J. L. (2011). Bayesian nonparametric modeling for causal inference. *Journal of Computational and Graphical Statistics* **20,** 217–240.

Ishwaran, H. and Kogalur, U. (2007). Random survival forests for r. *R News* **7,** 25–31.

Ishwaran, H. and Kogalur, U. (2019). *Fast Unified Random Forests for Survival, Regression, and Classification (RF-SRC).* R package version 2.9.1.

Ishwaran, H., Kogalur, U., Gorodeski, E., Minn, A., and Lauer, M. (2010). High-dimensional variable selection for survival data. *Journal of the American Statistical Association* **105,** 205–217.

Ishwaran, H., Kogalur, U. B., Blackstone, E. H., and Lauer, M. S. (2008). Random survival forests. *Annals of Applied Statistics* **2,** 841–860.

Lipkovich, I., Dmitrienko, A., and D'Agostino Sr., R. B. (2017). Tutorial in biostatistics:

data-driven subgroup identification and analysis in clinical trials. *Statistics in Medicine* **36,** 136–96.

Lu, M., Sadiq, S., Feaster, D. J., and Ishwaran, H. (2018). Estimating individual treatment effect in observational data using random forest methods. *Journal of Computational and Graphical Statistics* **27,** 209–19.

Lu, W., Zhang, H. H., and Zeng, D. (2013). Variable selection for optimal treatment decision. *Statist. Methods Med. Res.* **22,** 493–504.

Luedtke, A. R. and van der Laan, M. J. (2016). Statistical inference for the mean outcome under a possibly non-unique optimal treatment strategy. *Annals of Statistics* **44,** 713–42.

R Core Team (2019). *R: A Language and Environment for Statistical Computing.* R Foundation for Statistical Computing, Vienna, Austria.

R Core Team (2020). *R: A Language and Environment for Statistical Computing.* R Foundation for Statistical Computing, Vienna, Austria.

Rubin, D. (1974). Estimating causal effects of treatments in randomized and nonrandomized studies. *Journal of Educational Psychology* **66,** 688–701.

Therneau, T. and Atkinson, B. (2019). *rpart: Recursive Partitioning and Regression Trees.* R package version 4.1-15.

Tsiatis, A. A., Davidian, M., Zhang, M., and Lu, X. (2008). Covariate adjustment for two-sample treatment comparisons in randomized clinical trials: A principled yet flexible approach. *Statistics in Medicine* **27,** 4658–77.

Wager, S. and Athey, S. (2018). Estimation and inference of heterogeneous treatment effects using random forests. *Journal of the American Statistical Association* **113,** 1228–42.

Zhang, M., Tsiatis, A. A., and Davidian, M. (2008). Improving efficiency of inferences in randomized clinical trials using auxiliary covariates. *Biometrics* **64,** 707–15.

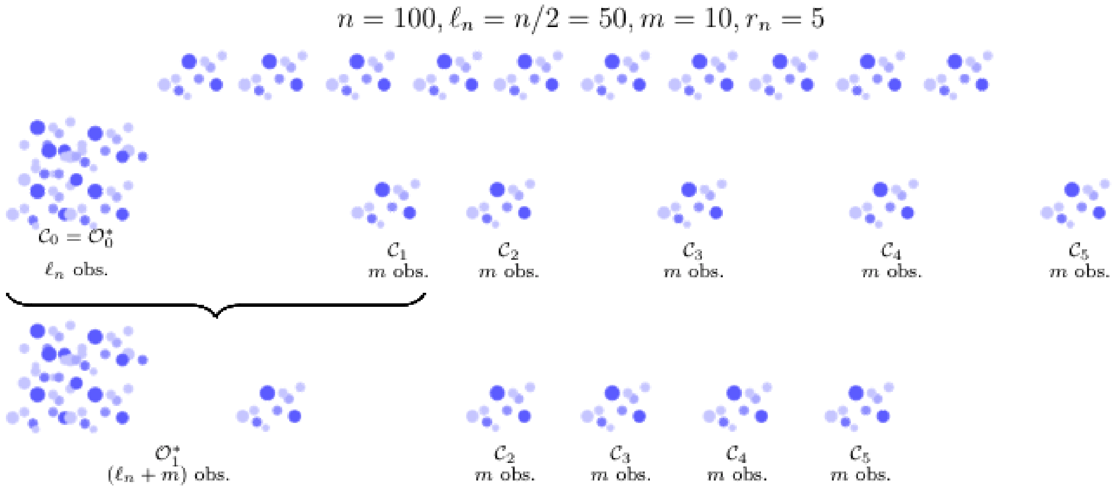

Figure 1. Diagram of how the data are partitioned into chunks. "obs." stands for observations.

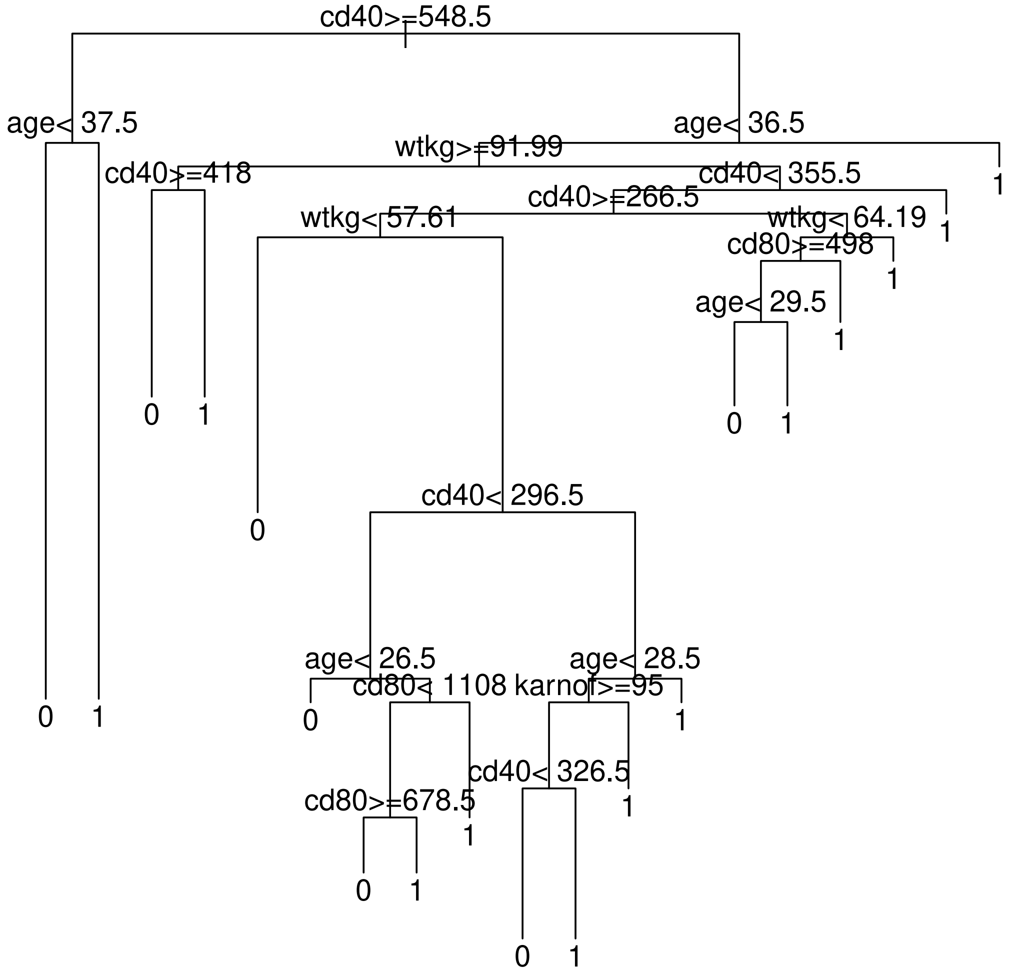

Figure 2. Classification tree for subgroup membership under the ACTG175 data analysis with zidovudine + didanosine as treatment. A subject is classified as '1' if s/he is expected to benefit from treatment (belong to subgroup), 0 otherwise.

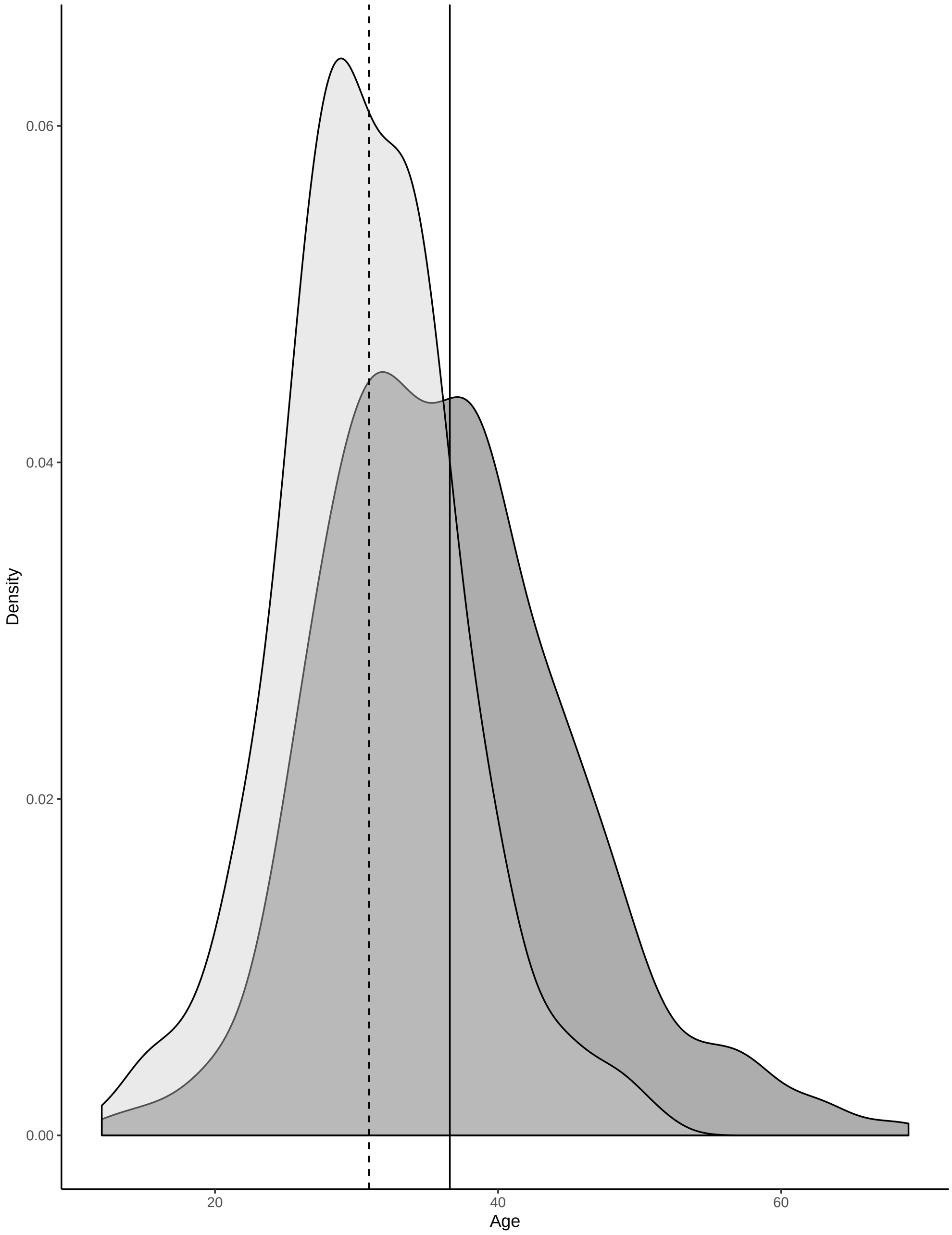

Figure 3. Density plot of age among patients predicted to benefit from zidovudine + didanosine (dark grey fill), and among patients predicted not to benefit from zidovudine + didanosine (light grey fill). Solid (dotted) vertical line corresponds to mean age within the group (not) predicted to benefit from zidovudine + didanosine.

Table 1

Model 1 randomized study results for $T_{IPW,\ SAP\text{-}match}$ computed using $\widehat{\sigma}_j$ in place of $\widehat{\sigma}_{j,m\pi}$ under scheme 1, $\pi = 0.5, \alpha = 0.05, n = 1000$. Mean ($\mu$) and standard deviation (σ) of the one-step value difference test statistic based on 500 simulated datasets. Largest standard error for μ and σ is 0.02 and 0.02 respectively.

c	μ	σ
0	0.01	0.39
-1	0.01	0.36

Table 2

Model 1 observational data results for $T_{IPW,\ SAP\text{-}match}$, under scheme 1, $\pi \approx 0.5$, $\alpha = 0.05, n = 1000$. Largest standard error for μ, σ, and $1-\beta\ /\alpha$ is $0.09, 0.28$, and 0.02 respectively.

c	μ	σ	$1-\beta\ /\alpha$
0.30	0.94	2.02	0.35
0.40	1.49	2.03	0.46
0.50	2.05	2.05	0.57
0.00	-0.33	2.02	0.16
-1.00	-0.38	2.11	0.18
-2.00	-0.36	2.10	0.17

Table 3

Model 1 randomized study results for $T_{AIPW,\ SBT}$ under scheme 2, $\pi = 0.5, \alpha = 0.05, n = 1000$. Largest standard error for μ, σ, and $1-\beta\ /\alpha$ is $0.05, 0.07$, and 0.02 respectively.

c	μ	σ	$1-\beta\ /\alpha$
0.30	2.27	1.02	0.74
0.40	3.21	0.98	0.95
0.50	4.15	0.97	0.99
0.00	0.05	1.03	0.06
-1.00	-0.12	0.98	0.04
-2.00	0.04	1.01	0.06

Table 4

Model 1 randomized study results under scheme 1, $\alpha = 0.05, n = 600$. Mean (μ), standard deviation (σ), and power or type I error ($1-\beta\ /\alpha$) of the one-step value difference test statistic based on 500 simulated datasets. Subscripts "IPW" and "AIPW" correspond to $T_{IPW,\bullet}$ and $T_{AIPW,\bullet}$, respectively. "Method" refers to chunking method, where "SBT" corresponds to method 1 and "SAP-match" corresponds to method 2. Largest standard error for μ, σ, and $1-\beta\ /\alpha$ is 0.05, 0.08, and 0.02 respectively.

c	method	μ_{IPW}	μ_{AIPW}	σ_{IPW}	σ_{AIPW}	$1-\beta_{\text{IPW}}$ $/\alpha_{\text{IPW}}$	$1-\beta_{\text{AIPW}}$ $/\alpha_{\text{AIPW}}$
$\pi = 0.4$							
0.30	SAP-match	1.26	1.80	0.98	1.01	0.31	0.57
0.30	SBT	0.63	1.75	1.00	1.02	0.15	0.53
0.40	SAP-match	1.78	2.48	0.97	0.99	0.55	0.80
0.40	SBT	0.89	2.44	1.00	1.01	0.23	0.80
0.50	SAP-match	2.32	3.18	0.97	1.00	0.73	0.92
0.50	SBT	1.16	3.16	1.00	1.02	0.33	0.93
0.00	SAP-match	0.02	0.05	0.98	1.00	0.06	0.04
0.00	SBT	0.06	0.02	0.99	0.98	0.06	0.04
-1.00	SAP-match	-0.18	-0.29	0.98	1.02	0.04	0.03
-1.00	SBT	-0.00	-0.22	0.96	0.99	0.04	0.02
-2.00	SAP-match	-0.07	-0.09	0.97	0.99	0.04	0.03
-2.00	SBT	0.03	-0.08	0.97	0.99	0.04	0.04
$\pi = 0.5$							
0.30	SAP-match	1.22	1.84	1.06	1.11	0.36	0.60
0.30	SBT	0.51	1.79	1.03	0.96	0.14	0.55
0.40	SAP-match	1.60	2.52	1.03	1.01	0.48	0.80
0.40	SBT	0.72	2.50	1.03	0.97	0.20	0.81
0.50	SAP-match	2.11	3.25	1.02	0.99	0.67	0.95
0.50	SBT	0.95	3.24	1.03	1.00	0.29	0.94
0.00	SAP-match	0.08	0.05	1.04	1.08	0.07	0.06
0.00	SBT	0.05	0.00	1.03	0.99	0.05	0.04
-1.00	SAP-match	-0.02	-0.16	1.02	1.02	0.05	0.03
-1.00	SBT	0.00	-0.22	1.01	1.02	0.05	0.03
-2.00	SAP-match	0.04	-0.04	1.02	1.01	0.07	0.05
-2.00	SBT	0.03	-0.05	1.02	1.01	0.06	0.05
$\pi = 0.6$							
0.30	SAP-match	0.88	1.73	1.06	1.00	0.24	0.53
0.30	SBT	0.33	1.71	1.01	0.98	0.09	0.54
0.40	SAP-match	1.32	2.44	1.04	0.99	0.37	0.77
0.40	SBT	0.51	2.44	1.01	0.97	0.14	0.79
0.50	SAP-match	1.81	3.20	1.03	0.95	0.54	0.95
0.50	SBT	0.69	3.17	1.01	0.96	0.19	0.94
0.00	SAP-match	-0.11	0.00	1.05	1.00	0.06	0.05
0.00	SBT	-0.04	0.00	1.00	0.95	0.05	0.03
-1.00	SAP-match	-0.20	-0.19	1.03	0.95	0.05	0.03
-1.00	SBT	-0.07	-0.17	0.99	0.96	0.04	0.04
-2.00	SAP-match	-0.15	-0.08	1.01	0.96	0.05	0.03
-2.00	SBT	-0.05	-0.07	0.99	0.99	0.04	0.04

Table 5

Model 1 randomized study results under scheme 1, $\alpha = 0.05, n = 1000$. Mean (μ), standard deviation (σ), and power or type I error ($1-\beta\ /\alpha$) of the one-step value difference test statistic based on 500 simulated datasets. Subscripts "IPW" and "AIPW" correspond to $T_{IPW,\bullet}$ and $T_{AIPW,\bullet}$, respectively. "Method" refers to chunking method, where "SBT" corresponds to method 1 and "SAP-match" corresponds to method 2. Largest standard error for μ, σ, and $1-\beta\ /\alpha$ is 0.05, 0.07, and 0.02 respectively.

c	method	μ_{IPW}	μ_{AIPW}	σ_{IPW}	σ_{AIPW}	$1-\beta_{\text{IPW}}\ /\alpha_{\text{IPW}}$	$1-\beta_{\text{AIPW}}\ /\alpha_{\text{AIPW}}$
$\pi = 0.4$							
0.30	SAP-match	1.70	2.31	0.97	1.02	0.52	0.74
0.30	SBT	0.74	2.35	1.00	0.97	0.18	0.78
0.40	SAP-match	2.43	3.27	0.96	1.04	0.81	0.94
0.40	SBT	1.10	3.32	1.00	0.96	0.30	0.96
0.50	SAP-match	3.17	4.23	0.96	1.02	0.94	1.00
0.50	SBT	1.45	4.25	1.00	0.98	0.41	1.00
0.00	SAP-match	-0.02	-0.01	0.96	1.01	0.04	0.06
0.00	SBT	-0.03	-0.01	1.01	0.95	0.05	0.05
-1.00	SAP-match	-0.07	-0.11	0.98	0.96	0.04	0.04
-1.00	SBT	-0.06	-0.15	1.01	0.99	0.05	0.04
-2.00	SAP-match	-0.03	-0.04	0.97	0.96	0.04	0.04
-2.00	SBT	-0.04	-0.05	1.01	0.98	0.05	0.04
$\pi = 0.5$							
0.30	SAP-match	1.61	2.50	1.01	1.01	0.51	0.80
0.30	SBT	0.62	2.55	1.00	0.99	0.15	0.81
0.40	SAP-match	2.29	3.39	1.01	1.00	0.73	0.96
0.40	SBT	0.92	3.44	1.00	1.01	0.21	0.97
0.50	SAP-match	2.92	4.40	0.94	1.02	0.92	1.00
0.50	SBT	1.17	4.43	1.00	1.01	0.30	1.00
0.00	SAP-match	0.03	-0.03	1.00	0.99	0.04	0.05
0.00	SBT	-0.03	0.02	1.01	1.00	0.06	0.06
-1.00	SAP-match	0.02	-0.06	0.99	1.00	0.04	0.05
-1.00	SBT	-0.05	-0.07	1.02	1.04	0.06	0.05
-2.00	SAP-match	0.03	-0.03	0.98	0.99	0.05	0.05
-2.00	SBT	-0.04	-0.00	1.02	1.03	0.06	0.06
$\pi = 0.6$							
0.30	SAP-match	1.45	2.34	0.97	1.03	0.42	0.76
0.30	SBT	0.57	2.41	1.02	0.99	0.14	0.78
0.40	SAP-match	2.06	3.34	0.95	1.00	0.68	0.96
0.40	SBT	0.82	3.38	1.01	0.96	0.20	0.96
0.50	SAP-match	2.59	4.33	0.98	0.98	0.83	1.00
0.50	SBT	1.04	4.38	1.02	0.98	0.27	1.00
0.00	SAP-match	0.07	0.01	0.97	0.97	0.06	0.04
0.00	SBT	0.05	0.06	1.01	0.95	0.05	0.04
-1.00	SAP-match	0.00	-0.15	0.95	0.91	0.05	0.03
-1.00	SBT	0.03	-0.06	1.01	1.00	0.05	0.04
-2.00	SAP-match	0.03	-0.09	0.94	0.92	0.04	0.04
-2.00	SBT	0.04	-0.02	1.01	0.99	0.05	0.05

Table 6

Model 2 randomized study results under scheme 1, $\alpha = 0.05, n = 600$. Mean (μ), standard deviation (σ), and power or type I error ($1 - \beta$ /α) of the one-step value difference test statistic based on 500 simulated datasets. Subscripts "IPW" and "AIPW" correspond to $T_{IPW,\bullet}$ and $T_{AIPW,\bullet}$, respectively. "Method" refers to chunking method, where "SBT" corresponds to method 1 and "SAP-match" corresponds to method 2. Largest standard error for μ, σ, and $1 - \beta$ /α is 0.05, 0.07, and 0.02 respectively.

c	method	μ_{IPW}	μ_{AIPW}	σ_{IPW}	σ_{AIPW}	$1-\beta_{\text{IPW}}$ /α_{IPW}	$1-\beta_{\text{AIPW}}$ /α_{AIPW}
$\pi = 0.4$							
1.20	SAP-match	1.76	2.31	1.01	1.01	0.56	0.75
1.20	SBT	1.46	2.35	1.02	0.94	0.41	0.79
1.30	SAP-match	1.91	2.49	1.00	1.00	0.63	0.81
1.30	SBT	1.59	2.53	1.03	0.95	0.47	0.82
1.40	SAP-match	2.04	2.64	0.99	1.00	0.67	0.86
1.40	SBT	1.72	2.70	1.03	0.95	0.52	0.86
0.00	SAP-match	0.07	0.03	0.99	1.01	0.05	0.07
0.00	SBT	0.02	0.02	1.04	1.00	0.08	0.06
-1.00	SAP-match	-0.06	-0.18	1.00	1.03	0.05	0.05
-1.00	SBT	-0.11	-0.21	1.04	0.97	0.05	0.04
-2.00	SAP-match	0.04	-0.02	0.98	1.04	0.05	0.06
-2.00	SBT	-0.04	-0.09	1.05	0.98	0.07	0.04
$\pi = 0.5$							
1.20	SAP-match	1.61	2.42	0.97	0.98	0.48	0.79
1.20	SBT	1.22	2.42	1.02	0.96	0.32	0.79
1.30	SAP-match	1.74	2.60	0.97	0.97	0.53	0.83
1.30	SBT	1.33	2.60	1.02	0.97	0.36	0.84
1.40	SAP-match	1.88	2.77	0.97	0.98	0.60	0.87
1.40	SBT	1.44	2.77	1.02	0.96	0.41	0.89
0.00	SAP-match	0.04	0.00	0.95	1.00	0.04	0.05
0.00	SBT	0.00	0.04	1.01	0.96	0.06	0.05
-1.00	SAP-match	-0.06	-0.18	0.97	0.98	0.03	0.03
-1.00	SBT	-0.11	-0.21	1.03	0.96	0.05	0.02
-2.00	SAP-match	0.01	-0.05	0.95	0.96	0.04	0.03
-2.00	SBT	-0.06	-0.10	1.03	0.96	0.05	0.04
$\pi = 0.6$							
1.20	SAP-match	1.45	2.41	1.01	0.97	0.41	0.79
1.20	SBT	1.06	2.43	1.00	0.93	0.28	0.80
1.30	SAP-match	1.57	2.59	1.01	0.97	0.46	0.84
1.30	SBT	1.15	2.61	0.99	0.93	0.31	0.85
1.40	SAP-match	1.69	2.75	1.01	0.96	0.51	0.87
1.40	SBT	1.25	2.78	0.99	0.93	0.35	0.90
0.00	SAP-match	0.07	0.02	1.00	0.99	0.05	0.07
0.00	SBT	0.02	0.05	1.00	0.95	0.04	0.05
-1.00	SAP-match	0.00	-0.13	1.01	0.97	0.05	0.03
-1.00	SBT	-0.04	-0.11	1.01	1.00	0.04	0.03
-2.00	SAP-match	0.05	-0.03	0.99	0.95	0.06	0.05
-2.00	SBT	-0.00	-0.01	1.01	0.97	0.04	0.04

Table 7

Model 2 randomized study results under scheme 1, $\alpha = 0.05, n = 1000$. Mean (μ), standard deviation (σ), and power or type I error ($1-\beta$ /α) of the one-step value difference test statistic based on 500 simulated datasets. Subscripts "IPW" and "AIPW" correspond to $T_{IPW,\bullet}$ and $T_{AIPW,\bullet}$, respectively. "Method" refers to chunking method, where "SBT" corresponds to method 1 and "SAP-match" corresponds to method 2. Largest standard error for μ, σ, and $1-\beta$ /α is 0.05, 0.08, and 0.02 respectively.

c	method	μ_{IPW}	μ_{AIPW}	σ_{IPW}	σ_{AIPW}	$1-\beta_{\text{IPW}}$ /α_{IPW}	$1-\beta_{\text{AIPW}}$ /α_{AIPW}
$\pi = 0.4$							
0.80	SAP-match	1.53	2.21	1.02	0.98	0.44	0.73
0.80	SBT	1.27	2.16	0.96	0.94	0.37	0.70
0.90	SAP-match	1.73	2.46	1.02	0.96	0.52	0.82
0.90	SBT	1.45	2.42	0.95	0.95	0.45	0.79
1.00	SAP-match	1.94	2.69	1.01	0.95	0.58	0.89
1.00	SBT	1.60	2.67	0.95	0.95	0.50	0.85
0.00	SAP-match	-0.00	0.08	0.97	1.03	0.04	0.06
0.00	SBT	0.02	-0.02	0.98	0.96	0.04	0.04
-1.00	SAP-match	-0.13	-0.11	0.98	1.07	0.04	0.06
-1.00	SBT	-0.04	-0.15	1.00	1.02	0.04	0.05
-2.00	SAP-match	-0.06	-0.00	0.99	1.07	0.04	0.07
-2.00	SBT	-0.01	-0.08	1.01	1.05	0.04	0.04
$\pi = 0.5$							
0.80	SAP-match	1.40	2.26	0.97	1.00	0.39	0.74
0.80	SBT	1.08	2.26	0.98	1.01	0.29	0.73
0.90	SAP-match	1.57	2.45	0.98	0.94	0.47	0.82
0.90	SBT	1.22	2.55	0.97	1.02	0.34	0.80
1.00	SAP-match	1.73	2.82	0.99	1.02	0.55	0.87
1.00	SBT	1.36	2.78	0.96	1.00	0.39	0.88
0.00	SAP-match	0.02	0.10	0.97	0.95	0.04	0.05
0.00	SBT	0.01	0.01	0.96	1.01	0.05	0.05
-1.00	SAP-match	-0.05	-0.02	0.98	0.93	0.03	0.03
-1.00	SBT	-0.05	-0.13	0.98	1.04	0.03	0.05
-2.00	SAP-match	-0.02	0.04	0.99	0.96	0.04	0.04
-2.00	SBT	-0.03	-0.08	0.98	1.08	0.04	0.06
$\pi = 0.6$							
0.80	SAP-match	1.18	2.20	1.01	1.00	0.32	0.71
0.80	SBT	0.86	2.15	1.02	1.02	0.23	0.71
0.90	SAP-match	1.33	2.48	0.97	1.01	0.36	0.82
0.90	SBT	0.98	2.43	1.02	1.03	0.25	0.78
1.00	SAP-match	1.50	2.73	1.02	1.00	0.44	0.87
1.00	SBT	1.10	2.67	1.04	1.04	0.29	0.83
0.00	SAP-match	-0.00	0.01	0.99	0.97	0.04	0.04
0.00	SBT	-0.02	0.00	1.02	1.10	0.06	0.06
-1.00	SAP-match	-0.06	-0.10	0.98	0.96	0.05	0.03
-1.00	SBT	-0.06	-0.13	1.02	1.07	0.06	0.04
-2.00	SAP-match	-0.01	-0.00	0.97	0.95	0.05	0.04
-2.00	SBT	-0.02	-0.01	1.02	1.08	0.06	0.05

Table 8

Model 3 randomized study results under scheme 1, $\alpha = 0.05, n = 600$. Mean (μ), standard deviation (σ), and power or type I error ($1-\beta\ /\alpha$) of the one-step value difference test statistic based on 500 simulated datasets. Subscripts "IPW" and "AIPW" correspond to $T_{IPW,\bullet}$ and $T_{AIPW,\bullet}$, respectively. "Method" refers to chunking method, where "SBT" corresponds to method 1 and "SAP-match" corresponds to method 2. Largest standard error for μ, σ, and $1-\beta\ /\alpha$ is 0.05, 0.07, and 0.02 respectively.

c	method	μ_{IPW}	μ_{AIPW}	σ_{IPW}	σ_{AIPW}	$1-\beta_{\text{IPW}}$ $/\alpha_{\text{IPW}}$	$1-\beta_{\text{AIPW}}$ $/\alpha_{\text{AIPW}}$
$\pi = 0.4$							
0.10	SAP-match	0.61	0.90	0.98	0.98	0.15	0.23
0.10	SBT	0.34	0.89	1.00	1.01	0.09	0.25
0.20	SAP-match	1.34	1.90	0.96	0.97	0.36	0.59
0.20	SBT	0.69	1.90	1.01	1.02	0.16	0.61
0.30	SAP-match	2.18	3.00	0.97	0.99	0.69	0.91
0.30	SBT	1.10	2.98	1.01	1.01	0.31	0.90
0.00	SAP-match	0.02	0.05	0.98	1.00	0.06	0.04
0.00	SBT	0.06	0.02	0.99	0.98	0.06	0.04
-1.00	SAP-match	-0.22	-0.50	0.98	0.97	0.03	0.02
-1.00	SBT	-0.02	-0.47	1.00	1.05	0.05	0.01
-2.00	SAP-match	-0.03	-0.02	0.99	0.99	0.06	0.06
-2.00	SBT	0.05	-0.08	0.99	0.99	0.05	0.04
$\pi = 0.5$							
0.10	SAP-match	0.51	0.90	1.02	1.00	0.13	0.23
0.10	SBT	0.27	0.91	1.03	0.98	0.09	0.24
0.20	SAP-match	1.18	1.91	1.04	1.04	0.32	0.59
0.20	SBT	0.56	1.94	1.03	0.97	0.15	0.62
0.30	SAP-match	1.95	3.01	1.06	1.04	0.60	0.91
0.30	SBT	0.90	3.05	1.03	1.02	0.26	0.91
0.00	SAP-match	-0.03	0.01	1.03	0.98	0.06	0.06
0.00	SBT	0.05	0.00	1.02	0.98	0.05	0.06
-1.00	SAP-match	-0.20	-0.44	1.04	1.02	0.05	0.02
-1.00	SBT	-0.01	-0.43	1.02	1.04	0.05	0.02
-2.00	SAP-match	-0.04	0.03	1.04	1.05	0.06	0.07
-2.00	SBT	0.04	-0.01	1.02	1.03	0.05	0.06
$\pi = 0.6$							
0.10	SAP-match	0.37	0.86	1.04	0.99	0.11	0.23
0.10	SBT	0.13	0.84	1.00	0.98	0.06	0.23
0.20	SAP-match	0.97	1.86	1.03	0.98	0.25	0.58
0.20	SBT	0.37	1.84	1.01	0.98	0.11	0.58
0.30	SAP-match	1.67	2.98	1.05	0.98	0.50	0.92
0.30	SBT	0.64	2.94	1.02	0.98	0.17	0.90
0.00	SAP-match	-0.11	0.00	1.05	1.00	0.06	0.05
0.00	SBT	-0.04	0.00	1.00	0.95	0.05	0.03
-1.00	SAP-match	-0.25	-0.45	1.03	1.03	0.04	0.02
-1.00	SBT	-0.08	-0.40	0.99	1.00	0.04	0.02
-2.00	SAP-match	-0.13	0.00	1.04	1.08	0.06	0.07
-2.00	SBT	-0.05	-0.02	0.99	1.00	0.04	0.05

Table 9

Model 3 randomized study results under scheme 1, $\alpha = 0.05, n = 1000$. Mean (μ), standard deviation (σ), and power or type I error ($1 - \beta$ /α) of the one-step value difference test statistic based on 500 simulated datasets. Subscripts "IPW" and "AIPW" correspond to $T_{IPW,\bullet}$ and $T_{AIPW,\bullet}$, respectively. "Method" refers to chunking method, where "SBT" corresponds to method 1 and "SAP-match" corresponds to method 2. Largest standard error for μ, σ, and $1 - \beta$ /α is 0.05, 0.07, and 0.02 respectively.

c	method	μ_{IPW}	μ_{AIPW}	σ_{IPW}	σ_{AIPW}	$1-\beta_{\text{IPW}}$ /α_{IPW}	$1-\beta_{\text{AIPW}}$ /α_{AIPW}
$\pi = 0.4$							
0.10	SAP-match	0.81	1.10	1.03	1.00	0.20	0.28
0.10	SBT	0.32	1.14	1.01	0.96	0.09	0.29
0.15	SAP-match	1.29	1.77	1.03	1.00	0.37	0.55
0.15	SBT	0.55	1.81	1.01	0.97	0.12	0.56
0.20	SAP-match	1.79	2.53	0.99	1.01	0.56	0.81
0.20	SBT	0.82	2.54	1.00	0.99	0.20	0.82
0.00	SAP-match	-0.02	-0.01	0.96	1.01	0.04	0.06
0.00	SBT	-0.03	-0.01	1.01	0.95	0.05	0.05
-1.00	SAP-match	-0.08	-0.20	0.96	0.99	0.03	0.03
-1.00	SBT	-0.06	-0.19	1.01	0.98	0.04	0.03
-2.00	SAP-match	-0.00	0.02	0.97	0.99	0.05	0.05
-2.00	SBT	-0.04	-0.06	1.01	1.04	0.05	0.05
$\pi = 0.5$							
0.10	SAP-match	0.75	1.17	0.94	1.05	0.18	0.32
0.10	SBT	0.26	1.25	1.00	0.96	0.08	0.33
0.15	SAP-match	1.19	1.83	0.95	1.03	0.31	0.59
0.15	SBT	0.44	1.89	1.00	0.97	0.10	0.58
0.20	SAP-match	1.70	2.62	1.02	1.03	0.54	0.84
0.20	SBT	0.67	2.66	1.00	0.98	0.15	0.85
0.00	SAP-match	0.03	-0.03	1.00	0.99	0.04	0.05
0.00	SBT	-0.03	0.02	1.01	1.00	0.06	0.06
-1.00	SAP-match	0.02	-0.11	0.97	1.00	0.05	0.03
-1.00	SBT	-0.05	-0.14	1.02	1.05	0.05	0.05
-2.00	SAP-match	0.06	0.04	0.98	1.01	0.06	0.06
-2.00	SBT	-0.04	-0.01	1.02	1.05	0.06	0.07
$\pi = 0.6$							
0.10	SAP-match	0.71	1.20	0.97	0.98	0.14	0.32
0.10	SBT	0.30	1.25	1.01	0.97	0.10	0.33
0.15	SAP-match	1.04	1.78	0.96	0.99	0.27	0.57
0.15	SBT	0.43	1.87	1.01	0.95	0.11	0.59
0.20	SAP-match	1.55	2.62	0.97	0.97	0.47	0.84
0.20	SBT	0.63	2.65	1.01	0.96	0.15	0.87
0.00	SAP-match	0.07	0.01	0.97	0.97	0.06	0.04
0.00	SBT	0.05	0.06	1.01	0.95	0.05	0.04
-1.00	SAP-match	0.02	-0.21	0.98	0.96	0.05	0.03
-1.00	SBT	0.03	-0.12	1.01	1.05	0.05	0.04
-2.00	SAP-match	0.06	-0.02	0.99	0.98	0.06	0.04
-2.00	SBT	0.04	0.02	1.01	1.03	0.05	0.06

Table 10

Model 1 observational study results under scheme 1, $\alpha = 0.05, n = 600$. Mean (μ), standard deviation (σ), and power or type I error ($1-\beta\ /\alpha$) of the one-step value difference test statistic based on 500 simulated datasets. Subscripts "I,SBT", "A,SAP" and "A,SBT" correspond to $T_{IPW,\ SBT}$, $T_{AIPW,SAP\text{-}match}$, and $T_{AIPW,SBT}$ respectively. Largest standard error for μ, σ, and $1-\beta\ /\alpha$ is $0.05, 0.07$, and 0.02 respectively.

c	$\mu_{\text{I, SBT}}$	$\mu_{\text{A, SAP}}$	$\mu_{\text{A, SBT}}$	$\sigma_{\text{I, SBT}}$	$\sigma_{\text{A, SBT}}$	$\sigma_{\text{A, SAP}}$	$1-\beta_{\text{I, SBT}}\ /\alpha_{\text{I, SBT}}$	$1-\beta_{\text{A, SAP}}\ /\alpha_{\text{A, SAP}}$	$1-\beta_{\text{A, SBT}}\ /\alpha_{\text{A, SBT}}$
$\pi \approx 0.4$									
0.30	0.37	1.63	1.61	1.07	1.05	1.03	0.11	0.49	0.49
0.40	0.62	2.29	2.30	1.08	1.05	1.05	0.17	0.72	0.71
0.50	0.88	2.99	2.99	1.09	1.05	1.03	0.25	0.90	0.90
0.00	-0.18	-0.05	-0.06	1.04	0.99	0.99	0.04	0.04	0.04
-1.00	-0.24	-0.32	-0.30	1.04	0.99	1.05	0.03	0.02	0.03
-2.00	-0.20	-0.12	-0.13	1.04	1.00	1.01	0.04	0.04	0.04
$\pi \approx 0.5$									
0.30	0.16	1.62	1.61	1.04	1.00	1.01	0.07	0.45	0.48
0.40	0.36	2.33	2.32	1.05	1.01	1.01	0.11	0.74	0.76
0.50	0.58	3.06	3.06	1.06	1.00	1.01	0.17	0.92	0.93
0.00	-0.29	-0.10	-0.13	1.03	0.99	0.98	0.04	0.05	0.04
-1.00	-0.30	-0.24	-0.24	1.02	0.96	0.97	0.03	0.04	0.04
-2.00	-0.28	-0.10	-0.12	1.02	0.95	0.96	0.03	0.03	0.03
$\pi \approx 0.6$									
0.30	0.07	1.61	1.62	1.03	0.98	1.00	0.07	0.48	0.48
0.40	0.22	2.29	2.26	1.04	1.00	0.99	0.09	0.73	0.75
0.50	0.40	3.00	2.98	1.04	1.00	1.01	0.11	0.92	0.92
0.00	-0.30	-0.11	-0.11	1.02	0.93	0.93	0.03	0.03	0.03
-1.00	-0.33	-0.26	-0.28	1.02	0.96	0.94	0.03	0.03	0.02
-2.00	-0.31	-0.13	-0.14	1.02	0.98	0.98	0.03	0.04	0.04

Table 11

Model 1 observational study results under scheme 1, $\alpha = 0.05, n = 1000$. Mean (μ), standard deviation (σ), and power or type I error ($1-\beta$ /α) of the one-step value difference test statistic based on 500 simulated datasets. Subscripts "I,SBT", "A,SAP" and "A,SBT" correspond to $T_{IPW,\ SBT}$, $T_{AIPW,SAP\text{-}match}$, and $T_{AIPW,SBT}$ respectively. Largest standard error for μ, σ, and $1-\beta$ /α is $0.05, 0.07$, and 0.02 respectively.

c	$\mu_{\text{I, SBT}}$	$\mu_{\text{A, SAP}}$	$\mu_{\text{A, SBT}}$	$\sigma_{\text{I, SBT}}$	$\sigma_{\text{A, SBT}}$	$\sigma_{\text{A, SAP}}$	$1-\beta_{\text{I, SBT}}/\alpha_{\text{I, SBT}}$	$1-\beta_{\text{A, SAP}}/\alpha_{\text{A, SAP}}$	$1-\beta_{\text{A, SBT}}/\alpha_{\text{A, SBT}}$
$\pi \approx 0.4$									
0.20	0.30	1.39	1.42	1.00	1.04	1.04	0.10	0.41	0.43
0.30	0.59	2.29	2.31	1.01	1.07	1.06	0.15	0.74	0.74
0.35	0.75	2.76	2.77	1.01	1.08	1.08	0.18	0.85	0.85
0.00	-0.14	-0.10	-0.04	0.99	1.02	1.01	0.04	0.05	0.04
-1.00	-0.16	-0.18	-0.14	0.99	1.04	1.01	0.04	0.03	0.02
-2.00	-0.16	-0.11	-0.10	0.99	1.02	1.00	0.03	0.04	0.04
$\pi \approx 0.5$									
0.20	0.17	1.54	1.52	0.99	1.01	0.98	0.07	0.45	0.46
0.30	0.43	2.46	2.46	1.00	1.02	1.00	0.13	0.81	0.80
0.35	0.54	2.86	2.89	1.00	1.00	1.01	0.14	0.89	0.90
0.00	-0.19	0.03	0.03	0.98	1.00	1.00	0.03	0.05	0.06
-1.00	-0.21	-0.09	-0.08	0.98	0.96	0.98	0.02	0.04	0.04
-2.00	-0.20	-0.02	0.01	0.98	0.96	0.97	0.03	0.05	0.06
$\pi \approx 0.6$									
0.20	0.12	1.44	1.41	1.00	1.03	1.04	0.05	0.41	0.40
0.30	0.31	2.33	2.31	1.00	1.03	1.02	0.08	0.74	0.74
0.35	0.42	2.82	2.79	1.00	1.03	1.02	0.11	0.88	0.87
0.00	-0.17	-0.01	-0.03	0.99	0.99	1.00	0.03	0.05	0.05
-1.00	-0.19	-0.13	-0.14	0.99	0.93	0.98	0.03	0.04	0.03
-2.00	-0.18	-0.05	-0.06	0.99	0.95	0.95	0.03	0.04	0.04

Table 12

Model 2 observational study results under scheme 1, $\alpha = 0.05, n = 600$. Mean (μ), standard deviation (σ), and power or type I error ($1 - \beta\ /\alpha$) of the one-step value difference test statistic based on 500 simulated datasets. Subscripts "I,SBT", "A,SAP" and "A,SBT" correspond to $T_{IPW,\ SBT}$, $T_{AIPW,SAP\text{-}match}$, and $T_{AIPW,SBT}$ respectively. Largest standard error for μ, σ, and $1 - \beta\ /\alpha$ is $0.05, 0.08,$ and 0.02 respectively.

c	$\mu_{\text{I, SBT}}$	$\mu_{\text{A, SAP}}$	$\mu_{\text{A, SBT}}$	$\sigma_{\text{I, SBT}}$	$\sigma_{\text{A, SBT}}$	$\sigma_{\text{A, SAP}}$	$1-\beta_{\text{I, SBT}}/\alpha_{\text{I, SBT}}$	$1-\beta_{\text{A, SAP}}/\alpha_{\text{A, SAP}}$	$1-\beta_{\text{A, SBT}}/\alpha_{\text{A, SBT}}$
$\pi \approx 0.4$									
1.30	1.23	2.32	2.33	1.08	1.06	1.10	0.36	0.73	0.74
1.40	1.34	2.48	2.49	1.08	1.06	1.08	0.41	0.79	0.78
1.50	1.44	2.63	2.63	1.08	1.06	1.07	0.44	0.82	0.82
0.00	-0.12	-0.13	-0.11	1.02	1.08	1.08	0.04	0.05	0.04
-1.00	-0.19	-0.32	-0.26	1.04	1.05	1.06	0.03	0.03	0.03
-2.00	-0.16	-0.20	-0.18	1.03	1.02	1.02	0.04	0.03	0.02
$\pi \approx 0.5$									
1.30	0.96	2.38	2.40	1.12	1.05	1.04	0.27	0.77	0.75
1.40	1.05	2.54	2.57	1.12	1.05	1.02	0.30	0.82	0.81
1.50	1.13	2.70	2.72	1.13	1.06	1.05	0.33	0.85	0.84
0.00	-0.21	-0.12	-0.15	1.07	1.05	1.06	0.04	0.04	0.05
-1.00	-0.28	-0.33	-0.33	1.08	1.04	1.03	0.04	0.02	0.02
-2.00	-0.24	-0.19	-0.23	1.07	1.04	1.03	0.04	0.03	0.03
$\pi \approx 0.6$									
1.30	0.72	2.29	2.27	1.10	1.03	1.06	0.21	0.75	0.75
1.40	0.80	2.45	2.44	1.10	1.03	1.06	0.21	0.80	0.78
1.50	0.88	2.62	2.59	1.10	1.03	1.07	0.25	0.84	0.83
0.00	-0.23	-0.09	-0.13	1.07	1.05	1.05	0.04	0.04	0.04
-1.00	-0.28	-0.29	-0.27	1.07	1.05	1.06	0.04	0.02	0.03
-2.00	-0.26	-0.21	-0.21	1.08	1.02	1.04	0.03	0.03	0.02

Table 13

Model 2 observational study results under scheme 1, $\alpha = 0.05, n = 1000$. Mean (μ), standard deviation (σ), and power or type I error ($1 - \beta\ /\alpha$) of the one-step value difference test statistic based on 500 simulated datasets. Subscripts "I,SBT", "A,SAP" and "A,SBT" correspond to $T_{IPW,\ SBT}$, $T_{AIPW,SAP\text{-}match}$, and $T_{AIPW,SBT}$ respectively. Largest standard error for μ, σ, and $1 - \beta\ /\alpha$ is 0.05, 0.08, and 0.02 respectively.

c	$\mu_{\text{I, SBT}}$	$\mu_{\text{A, SAP}}$	$\mu_{\text{A, SBT}}$	$\sigma_{\text{I, SBT}}$	$\sigma_{\text{A, SBT}}$	$\sigma_{\text{A, SAP}}$	$1 - \beta_{\text{I, SBT}} / \alpha_{\text{I, SBT}}$	$1 - \beta_{\text{A, SAP}} / \alpha_{\text{A, SAP}}$	$1 - \beta_{\text{A, SBT}} / \alpha_{\text{A, SBT}}$
$\pi \approx 0.4$									
0.80	0.99	2.06	2.06	1.06	1.01	1.00	0.27	0.65	0.66
0.90	1.13	2.31	2.32	1.06	0.99	1.00	0.30	0.76	0.76
1.00	1.28	2.55	2.55	1.06	0.98	0.99	0.35	0.81	0.84
0.00	-0.10	-0.04	-0.06	1.02	0.99	0.99	0.03	0.04	0.04
-1.00	-0.16	-0.21	-0.21	1.02	1.01	1.00	0.03	0.03	0.03
-2.00	-0.13	-0.15	-0.15	1.04	1.02	1.03	0.04	0.03	0.03
$\pi \approx 0.5$									
0.80	0.71	2.01	2.01	1.10	1.10	1.10	0.18	0.62	0.63
0.90	0.83	2.29	2.31	1.11	1.11	1.10	0.22	0.71	0.74
1.00	0.94	2.52	2.52	1.12	1.11	1.10	0.26	0.79	0.78
0.00	-0.18	-0.12	-0.10	1.06	1.04	1.07	0.04	0.05	0.06
-1.00	-0.21	-0.24	-0.19	1.04	1.06	1.06	0.04	0.03	0.03
-2.00	-0.19	-0.15	-0.14	1.05	1.09	1.06	0.04	0.05	0.05
$\pi \approx 0.6$									
0.80	0.63	2.03	2.02	1.03	1.03	1.05	0.16	0.63	0.63
0.90	0.73	2.29	2.27	1.03	1.04	1.03	0.19	0.72	0.73
1.00	0.84	2.54	2.52	1.03	1.05	1.04	0.21	0.80	0.79
0.00	-0.12	-0.04	-0.04	1.00	1.05	1.10	0.04	0.06	0.06
-1.00	-0.16	-0.15	-0.15	0.99	1.00	1.05	0.04	0.04	0.05
-2.00	-0.14	-0.06	-0.08	0.98	1.05	1.04	0.04	0.06	0.04

Table 14

Model 3 observational study results under scheme 1, $\alpha = 0.05, n = 600$. Mean (μ), standard deviation (σ), and power or type I error ($1-\beta\ /\alpha$) of the one-step value difference test statistic based on 500 simulated datasets. Subscripts "I,SBT", "A,SAP" and "A,SBT" correspond to $T_{IPW,\ SBT}$, $T_{AIPW,SAP\text{-}match}$, and $T_{AIPW,SBT}$ respectively. Largest standard error for μ, σ, and $1-\beta\ /\alpha$ is $0.05, 0.07,$ and 0.02 respectively.

c	$\mu_{\text{I, SBT}}$	$\mu_{\text{A, SAP}}$	$\mu_{\text{A, SBT}}$	$\sigma_{\text{I, SBT}}$	$\sigma_{\text{A, SBT}}$	$\sigma_{\text{A, SAP}}$	$1-\beta_{\text{I, SBT}}\ /\alpha_{\text{I, SBT}}$	$1-\beta_{\text{A, SAP}}\ /\alpha_{\text{A, SAP}}$	$1-\beta_{\text{A, SBT}}\ /\alpha_{\text{A, SBT}}$
$\pi \approx 0.4$									
0.10	0.09	0.77	0.79	1.05	1.02	1.02	0.06	0.19	0.19
0.20	0.43	1.77	1.78	1.06	1.07	1.06	0.13	0.54	0.53
0.30	0.83	2.87	2.87	1.07	1.08	1.09	0.24	0.88	0.88
0.00	-0.18	-0.05	-0.06	1.04	0.99	0.99	0.04	0.04	0.04
-1.00	-0.25	-0.57	-0.52	1.04	1.02	0.99	0.04	0.01	0.01
-2.00	-0.17	-0.03	-0.03	1.05	1.05	1.03	0.04	0.06	0.05
$\pi \approx 0.5$									
0.10	-0.07	0.77	0.76	1.03	0.98	0.99	0.05	0.19	0.18
0.20	0.20	1.77	1.77	1.05	0.99	1.02	0.09	0.57	0.54
0.30	0.53	2.89	2.87	1.06	1.00	1.03	0.16	0.90	0.90
0.00	-0.28	-0.12	-0.10	1.03	0.98	1.00	0.04	0.04	0.04
-1.00	-0.32	-0.44	-0.49	1.03	1.06	1.01	0.04	0.03	0.01
-2.00	-0.27	-0.03	-0.06	1.02	1.05	1.04	0.04	0.07	0.04
$\pi \approx 0.6$									
0.10	-0.13	0.74	0.72	1.03	0.96	0.98	0.04	0.19	0.17
0.20	0.09	1.72	1.71	1.03	0.98	0.98	0.06	0.50	0.52
0.30	0.36	2.79	2.80	1.05	1.01	1.01	0.11	0.87	0.88
0.00	-0.30	-0.11	-0.11	1.02	0.93	0.93	0.03	0.03	0.03
-1.00	-0.33	-0.43	-0.46	1.01	1.02	1.02	0.03	0.02	0.02
-2.00	-0.29	-0.01	-0.02	1.01	1.01	1.03	0.03	0.05	0.05

Table 15

Model 3 observational study results under scheme 1, $\alpha = 0.05, n = 1000$. Mean (μ), standard deviation (σ), and power or type I error ($1-\beta\ /\alpha$) of the one-step value difference test statistic based on 500 simulated datasets. Subscripts "I,SBT", "A,SAP" and "A,SBT" correspond to $T_{IPW,\ SBT}$, $T_{AIPW,SAP\text{-}match}$, and $T_{AIPW,SBT}$ respectively. Largest standard error for μ, σ, and $1-\beta\ /\alpha$ is $0.05, 0.07,$ and 0.02 respectively.

c	$\mu_{\text{I, SBT}}$	$\mu_{\text{A, SAP}}$	$\mu_{\text{A, SBT}}$	$\sigma_{\text{I, SBT}}$	$\sigma_{\text{A, SBT}}$	$\sigma_{\text{A, SAP}}$	$1-\beta_{\text{I, SBT}}/\alpha_{\text{I, SBT}}$	$1-\beta_{\text{A, SAP}}/\alpha_{\text{A, SAP}}$	$1-\beta_{\text{A, SBT}}/\alpha_{\text{A, SBT}}$
$\pi \approx 0.4$									
0.05	0.03	0.49	0.54	1.00	1.02	1.04	0.06	0.13	0.14
0.10	0.22	1.08	1.14	1.00	1.04	1.04	0.09	0.29	0.31
0.20	0.67	2.44	2.49	1.00	1.06	1.05	0.16	0.78	0.80
0.00	-0.14	-0.10	-0.04	0.99	1.02	1.01	0.04	0.05	0.04
-1.00	-0.16	-0.21	-0.23	1.01	1.01	0.99	0.04	0.03	0.03
-2.00	-0.14	-0.07	-0.08	1.02	1.03	1.04	0.04	0.05	0.05
$\pi \approx 0.5$									
0.05	-0.06	0.59	0.58	0.99	1.00	1.01	0.04	0.16	0.15
0.10	0.09	1.22	1.20	1.00	0.98	1.02	0.06	0.34	0.34
0.20	0.46	2.62	2.63	1.00	1.01	1.03	0.13	0.85	0.85
0.00	-0.19	0.03	0.03	0.98	1.00	1.00	0.03	0.05	0.06
-1.00	-0.22	-0.22	-0.18	0.97	0.98	1.00	0.02	0.02	0.03
-2.00	-0.20	-0.05	-0.02	0.98	1.02	1.00	0.02	0.05	0.05
$\pi \approx 0.6$									
0.05	-0.07	0.52	0.51	1.00	1.02	1.03	0.04	0.15	0.16
0.10	0.05	1.13	1.11	1.00	1.01	1.02	0.05	0.31	0.31
0.20	0.35	2.52	2.49	1.00	1.02	1.04	0.10	0.79	0.78
0.00	-0.17	-0.01	-0.03	0.99	0.99	1.00	0.03	0.05	0.05
-1.00	-0.18	-0.16	-0.12	0.99	1.02	1.06	0.03	0.04	0.04
-2.00	-0.17	0.03	-0.02	1.00	0.98	1.02	0.03	0.04	0.04

Table 16

Estimated range of % censoring before time L for $c = \{-1, -0.5, 0, 0.5, 0.75, 1.25\}$ *in the models listed above. Estimates based on 10,000 Monte Carlo samples from the model of interest.*

Model	L	% censoring before L
1a	30	31-43
1b	42	18-27
1c	33	33-44
2a	30	26-37
2b	42	15-23
2c	33	27-37

Table 17

Results under high-dimensional version (scheme 2) of Model 1c, with $\pi(\mathbf{X}_i) = 0.5$ and $L = 33$. Mean (μ), standard deviation (σ), and power or type I error ($1-\beta$ /α) of the one-step value difference test statistic T_{CAIPW}, is based on 500 simulated data sets of $n = \{600, 1000\}$. Largest standard error for μ and σ, and $1-\beta$ /α is 0.05 and 0.08, and 0.02, respectively.

c	n	μ_{CAIPW}	σ_{CAIPW}	$1-\beta_{\text{CAIPW}}$ /α_{CAIPW}
1.25	600	4.56	1.08	1.00
1.25	1000	6.18	1.06	1.00
0.75	600	3.08	1.06	0.91
0.75	1000	4.15	1.06	0.99
0.50	600	2.10	1.10	0.65
0.50	1000	2.84	1.07	0.88
0.00	600	-0.02	1.07	0.05
0.00	1000	0.01	1.00	0.05
-0.50	600	-0.14	1.10	0.03
-0.50	1000	-0.17	0.96	0.03
-1.00	600	-0.04	0.99	0.05
-1.00	1000	-0.05	0.99	0.05

Table 18

Results under Model 1a, with $\pi(\mathbf{X}_i) = 0.5$ and $L = 30$. Mean (μ), standard deviation (σ), and power or type I error ($1-\beta$ /α) of the one-step value difference test statistic is based on 500 simulated data sets of $n = \{600, 1000\}$. Subscripts "IPW", "AIPW", and "CAIPW" specify whether the results are baesd on T_{IPW}, T_{AIPW}, or T_{CAIPW}, respectively. Largest standard error for μ, σ, and $1-\beta$ /α is .06 and 0.11, and 0.02, respectively.

c	n	μ_{IPW}	μ_{AIPW}	μ_{CAIPW}	σ_{IPW}	σ_{AIPW}	σ_{CAIPW}	$1-\beta_{\text{IPW}}$ /α_{IPW}	$1-\beta_{\text{AIPW}}$ /α_{AIPW}	$1-\beta_{\text{CAIPW}}$ /α_{CAIPW}
1.25	600	1.96	1.97	4.20	1.02	1.03	1.31	0.62	0.61	0.97
1.25	1000	2.60	2.64	6.29	1.05	1.04	1.07	0.83	0.82	1.00
0.75	600	1.44	1.45	3.17	1.03	1.02	1.05	0.41	0.43	0.93
0.75	1000	1.89	1.93	4.31	1.04	1.04	1.03	0.61	0.61	0.99
0.50	600	1.04	1.07	2.17	1.05	1.05	1.04	0.30	0.31	0.70
0.50	1000	1.33	1.37	2.96	1.04	1.04	0.99	0.39	0.39	0.91
0.00	600	-0.01	-0.01	0.01	1.07	1.06	1.00	0.06	0.07	0.06
0.00	1000	-0.06	-0.04	0.06	1.05	1.03	1.05	0.05	0.06	0.07
-0.50	600	-0.17	-0.21	-0.25	1.07	1.07	1.01	0.04	0.04	0.02
-0.50	1000	-0.19	-0.19	-0.18	1.03	1.03	1.05	0.03	0.05	0.04
-1.00	600	-0.06	-0.06	-0.05	1.08	1.03	1.02	0.06	0.05	0.05
-1.00	1000	-0.07	-0.10	-0.08	1.02	1.04	1.04	0.03	0.05	0.04

Table 19

Results under Model 1b, with $\pi(\mathbf{X}_i) = 0.5$ and $L = 42$. Mean (μ), standard deviation (σ), and power or type I error ($1 - \beta\ /\alpha$) of the one-step value difference test statistic is based on 500 simulated data sets of $n = \{600, 1000\}$. Subscripts IPW, AIPW, and CAIPW specify whether the results are baesd on T_{IPW}, T_{AIPW}, or T_{CAIPW}, respectively. Largest standard error for μ and σ, and $1 - \beta\ /\alpha$ is .05 and 0.08, and 0.02, respectively.

c	n	μ_{IPW}	μ_{AIPW}	μ_{CAIPW}	σ_{IPW}	σ_{AIPW}	σ_{CAIPW}	$1 - \beta_{\text{IPW}}$ $/\alpha_{\text{IPW}}$	$1 - \beta_{\text{AIPW}}$ $/\alpha_{\text{AIPW}}$	$1 - \beta_{\text{CAIPW}}$ $/\alpha_{\text{CAIPW}}$
1.25	600	3.22	3.28	5.84	1.02	1.03	1.11	0.94	0.94	1.00
1.25	1000	4.16	4.25	7.76	1.04	1.02	1.08	1.00	0.99	1.00
0.75	600	2.18	2.24	3.71	1.04	1.05	1.05	0.70	0.72	0.98
0.75	1000	2.80	2.89	5.00	1.04	1.03	1.04	0.87	0.89	1.00
0.50	600	1.50	1.54	2.44	1.03	1.06	1.04	0.45	0.46	0.78
0.50	1000	1.95	2.02	3.35	1.04	1.04	1.00	0.62	0.65	0.96
0.00	600	0.05	0.05	-0.02	1.03	1.05	1.04	0.05	0.06	0.05
0.00	1000	0.00	0.04	0.05	1.01	1.03	1.08	0.05	0.05	0.08
-0.50	600	-0.14	-0.18	-0.23	1.08	1.03	1.01	0.05	0.02	0.03
-0.50	1000	-0.16	-0.18	-0.19	1.06	1.04	1.03	0.03	0.04	0.04
-1.00	600	-0.05	-0.07	-0.07	1.08	1.01	1.00	0.06	0.05	0.04
-1.00	1000	-0.04	-0.06	-0.07	1.04	1.02	1.03	0.04	0.05	0.05

Table 20

Results under Model 1c, with $\pi(\mathbf{X}_i) = 0.5$ and $L = 33$. Mean (μ), standard deviation (σ), and power or type I error ($1 - \beta$ /α) of the one-step value difference test statistic is based on 500 simulated data sets of $n = \{600, 1000\}$. Subscripts IPW, AIPW, and CAIPW specify whether the results are baesd on T_{IPW}, T_{AIPW}, or T_{CAIPW}, respectively. Largest standard error for μ and σ, and $1 - \beta$ /α is .05 and 0.08, and 0.02, respectively.

c	n	μ_{IPW}	μ_{AIPW}	μ_{CAIPW}	σ_{IPW}	σ_{AIPW}	σ_{CAIPW}	$1 - \beta_{\text{IPW}}$ /α_{IPW}	$1 - \beta_{\text{AIPW}}$ /α_{AIPW}	$1 - \beta_{\text{CAIPW}}$ /α_{CAIPW}
1.25	600	2.12	2.15	4.97	1.07	1.07	1.09	0.67	0.68	1.00
1.25	1000	2.76	2.81	6.63	1.06	1.03	1.07	0.85	0.86	1.00
0.75	600	1.46	1.48	3.27	1.06	1.05	1.04	0.44	0.44	0.95
0.75	1000	1.93	1.98	4.46	1.08	1.05	1.07	0.62	0.64	1.00
0.50	600	1.00	1.00	2.18	1.06	1.07	1.02	0.28	0.27	0.73
0.50	1000	1.33	1.38	3.01	1.09	1.07	1.05	0.37	0.42	0.90
0.00	600	0.01	-0.00	-0.02	1.07	1.02	0.99	0.07	0.05	0.05
0.00	1000	-0.09	-0.07	0.04	1.09	1.04	1.05	0.05	0.04	0.06
-0.50	600	-0.17	-0.19	-0.23	1.13	1.09	0.99	0.04	0.05	0.03
-0.50	1000	-0.16	-0.16	-0.20	1.05	0.98	1.03	0.04	0.03	0.04
-1.00	600	-0.07	-0.08	-0.07	1.12	1.06	1.00	0.06	0.06	0.06
-1.00	1000	-0.07	-0.08	-0.07	1.03	0.98	1.00	0.05	0.04	0.05

Table 21

Results under Model 2a, with $\pi(\mathbf{X}_i) = 0.5$ and $L = 30$. Mean (μ), standard deviation (σ), and power or type I error ($1 - \beta\ /\alpha$) of the one-step value difference test statistic is based on 500 simulated data sets of $n = \{600, 1000\}$. Subscripts "IPW", "AIPW", and "CAIPW" specify whether the results are baesd on T_{IPW}, T_{AIPW}, or T_{CAIPW}, respectively. Largest standard error for μ, σ, and $1 - \beta\ /\alpha$ is .05 and 0.08, and 0.02, respectively.

c	n	μ_{IPW}	μ_{AIPW}	μ_{CAIPW}	σ_{IPW}	σ_{AIPW}	σ_{CAIPW}	$1 - \beta_{\text{IPW}}\ /\alpha_{\text{IPW}}$	$1 - \beta_{\text{AIPW}}\ /\alpha_{\text{AIPW}}$	$1 - \beta_{\text{CAIPW}}\ /\alpha_{\text{CAIPW}}$
1.25	600	1.74	1.76	3.79	1.07	1.08	1.07	0.53	0.52	0.97
1.25	1000	2.37	2.36	4.93	1.00	1.02	1.05	0.76	0.77	1.00
0.75	600	1.10	1.11	2.28	1.11	1.12	1.06	0.31	0.31	0.72
0.75	1000	1.56	1.56	3.02	0.99	1.02	1.07	0.49	0.48	0.90
0.50	600	0.68	0.67	1.44	1.07	1.10	1.06	0.17	0.20	0.40
0.50	1000	1.04	1.02	1.90	1.00	1.00	1.04	0.28	0.28	0.59
0.00	600	-0.11	-0.13	-0.05	1.03	1.03	1.02	0.06	0.05	0.04
0.00	1000	0.00	0.00	-0.04	1.03	1.05	1.02	0.04	0.05	0.04
-0.50	600	-0.29	-0.28	-0.36	1.04	1.05	1.01	0.03	0.04	0.03
-0.50	1000	-0.25	-0.21	-0.36	1.03	1.00	0.97	0.04	0.03	0.02
-1.00	600	-0.20	-0.17	-0.14	1.01	1.02	0.99	0.03	0.04	0.03
-1.00	1000	-0.11	-0.06	-0.09	1.01	1.02	1.02	0.05	0.05	0.05

Table 22

Results under Model 2b, with $\pi(\mathbf{X}_i) = 0.5$ and $L = 42$. Mean (μ), standard deviation (σ), and power or type I error ($1-\beta$ /α) of the one-step value difference test statistic is based on 500 simulated data sets of $n = \{600, 1000\}$. Subscripts "IPW", "AIPW", and "CAIPW" specify whether the results are baesd on T_{IPW}, T_{AIPW}, or T_{CAIPW}, respectively. Largest standard error for μ, σ, and $1-\beta$ /α is .05 and 0.08, and 0.02, respectively.

c	n	μ_{IPW}	μ_{AIPW}	μ_{CAIPW}	σ_{IPW}	σ_{AIPW}	σ_{CAIPW}	$1-\beta_{\text{IPW}}$ /α_{IPW}	$1-\beta_{\text{AIPW}}$ /α_{AIPW}	$1-\beta_{\text{CAIPW}}$ /α_{CAIPW}
1.25	600	2.56	2.60	4.37	1.01	1.04	1.06	0.81	0.82	1.00
1.25	1000	3.43	3.44	5.68	0.98	1.01	1.10	0.97	0.96	1.00
0.75	600	1.57	1.59	2.61	1.01	1.02	1.02	0.47	0.45	0.85
0.75	1000	2.17	2.17	3.42	1.00	1.01	1.09	0.70	0.69	0.95
0.50	600	0.97	0.97	1.62	1.00	1.04	1.06	0.26	0.25	0.49
0.50	1000	1.42	1.41	2.16	0.99	0.99	1.08	0.42	0.41	0.69
0.00	600	-0.10	-0.11	-0.02	1.00	1.00	0.97	0.04	0.05	0.03
0.00	1000	-0.03	-0.05	-0.05	0.96	1.02	1.04	0.04	0.05	0.04
-0.50	600	-0.35	-0.34	-0.35	1.01	1.03	1.01	0.02	0.04	0.02
-0.50	1000	-0.31	-0.26	-0.34	0.96	0.96	0.94	0.02	0.03	0.02
-1.00	600	-0.17	-0.14	-0.14	1.05	1.02	1.00	0.04	0.03	0.03
-1.00	1000	-0.13	-0.06	-0.07	0.99	1.01	1.01	0.03	0.04	0.04

Table 23

Results under Model 2c, with $\pi(\mathbf{X}_i) = 0.5$ and $L = 33$. Mean (μ), standard deviation (σ), and power or type I error ($1-\beta$ $/\alpha$) of the one-step value difference test statistic is based on 500 simulated data sets of $n = \{600, 1000\}$. Subscripts "IPW", "AIPW", and "CAIPW" specify whether the results are baesd on T_{IPW}, T_{AIPW}, or T_{CAIPW}, respectively. Largest standard error for μ, σ, and $1-\beta$ $/\alpha$ is .05 and 0.08, and 0.02, respectively.

c	n	μ_{IPW}	μ_{AIPW}	μ_{CAIPW}	σ_{IPW}	σ_{AIPW}	σ_{CAIPW}	$1-\beta_{\text{IPW}}$ $/\alpha_{\text{IPW}}$	$1-\beta_{\text{AIPW}}$ $/\alpha_{\text{AIPW}}$	$1-\beta_{\text{CAIPW}}$ $/\alpha_{\text{CAIPW}}$
1.25	600	1.91	1.92	3.91	1.12	1.09	1.05	0.60	0.61	0.99
1.25	1000	2.56	2.56	5.04	1.05	1.04	1.07	0.80	0.80	1.00
0.75	600	1.22	1.22	2.35	1.12	1.10	1.02	0.36	0.36	0.75
0.75	1000	1.63	1.64	3.05	1.03	1.01	1.07	0.49	0.50	0.92
0.50	600	0.79	0.78	1.50	1.13	1.10	1.03	0.22	0.22	0.45
0.50	1000	1.10	1.10	1.93	1.05	1.04	1.06	0.30	0.30	0.62
0.00	600	-0.08	-0.08	-0.05	1.11	1.08	1.02	0.05	0.05	0.05
0.00	1000	0.02	-0.01	-0.07	1.03	1.02	0.99	0.06	0.05	0.03
-0.50	600	-0.29	-0.30	-0.40	1.02	0.98	1.01	0.02	0.03	0.02
-0.50	1000	-0.27	-0.27	-0.42	0.96	1.00	1.00	0.02	0.04	0.02
-1.00	600	-0.20	-0.17	-0.15	1.08	1.09	1.04	0.04	0.04	0.04
-1.00	1000	-0.15	-0.11	-0.12	0.99	1.07	1.04	0.04	0.05	0.04

Table 24

Results under Model 1a, with $\pi(\mathbf{X}_i) = exp(-0.3 + 0.2X_{1i} + 0.6X_{5i})/\{1 + exp(-0.3 + 0.2X_{1i} + 0.6X_{5i})\}$ and $L = 30$. Mean (μ), standard deviation (σ), and power or type I error ($1 - \beta$ /α) of the one-step value difference test statistic is based on 500 simulated data sets of $n = \{600, 1000\}$. Subscripts "IPW", "AIPW", and "CAIPW" specify whether the results are baesd on T_{IPW}, T_{AIPW}, or T_{CAIPW}, respectively. Largest standard error for μ, σ, and $1 - \beta$ /α is .05 and 0.08, and 0.02, respectively.

c	n	μ_{IPW}	μ_{AIPW}	μ_{CAIPW}	σ_{IPW}	σ_{AIPW}	σ_{CAIPW}	$1-\beta_{\text{IPW}}$ /α_{IPW}	$1-\beta_{\text{AIPW}}$ /α_{AIPW}	$1-\beta_{\text{CAIPW}}$ /α_{CAIPW}
1.25	600	1.75	1.90	4.64	1.05	1.08	1.08	0.56	0.60	1.00
1.25	1000	2.39	2.50	6.10	1.03	1.06	1.11	0.77	0.79	1.00
0.75	600	1.23	1.37	3.13	1.07	1.06	1.05	0.35	0.40	0.93
0.75	1000	1.69	1.79	4.15	1.00	1.01	1.05	0.50	0.55	1.00
0.50	600	0.84	0.97	2.09	1.05	1.04	1.03	0.22	0.26	0.66
0.50	1000	1.15	1.26	2.83	1.02	1.04	1.01	0.30	0.34	0.88
0.00	600	-0.21	-0.07	-0.05	1.09	1.06	0.99	0.04	0.05	0.04
0.00	1000	-0.13	-0.04	-0.05	1.02	1.01	0.97	0.03	0.06	0.05
-0.50	600	-0.32	-0.16	-0.26	1.09	1.06	0.99	0.03	0.04	0.03
-0.50	1000	-0.28	-0.11	-0.18	1.03	1.06	1.07	0.03	0.04	0.05
-1.00	600	-0.26	-0.08	-0.12	1.08	1.05	1.06	0.03	0.05	0.05
-1.00	1000	-0.19	0.05	0.04	1.00	1.00	1.00	0.03	0.06	0.05

Table 25

Results under Model 2a, with $\pi(\mathbf{X}_i) = exp(-0.3 + 0.2X_{1i} + 0.6X_{5i})/\{1 + exp(-0.3 + 0.2X_{1i} + 0.6X_{5i})\}$ and $L = 30$. Mean (μ), standard deviation (σ), and power or type I error ($1 - \beta$ /α) of the one-step value difference test statistic is based on 500 simulated data sets of $n = \{600, 1000\}$. Subscripts "IPW", "AIPW", and "CAIPW" specify whether the results are baesd on T_{IPW}, T_{AIPW}, or T_{CAIPW}, respectively. Largest standard error for μ, σ, and $1 - \beta$ /α is .05 and 0.08, and 0.02, respectively.

c	n	μ_{IPW}	μ_{AIPW}	μ_{CAIPW}	σ_{IPW}	σ_{AIPW}	σ_{CAIPW}	$1 - \beta_{\text{IPW}}$ /α_{IPW}	$1 - \beta_{\text{AIPW}}$ /α_{AIPW}	$1 - \beta_{\text{CAIPW}}$ /α_{CAIPW}
1.25	600	1.67	1.78	3.68	1.08	1.03	1.02	0.50	0.55	0.97
1.25	1000	2.21	2.32	5.04	1.01	1.01	1.04	0.71	0.75	1.00
0.75	600	1.07	1.17	2.22	1.06	1.01	1.00	0.30	0.31	0.71
0.75	1000	1.44	1.55	3.19	1.05	1.03	1.03	0.40	0.47	0.94
0.50	600	0.67	0.78	1.42	1.04	0.99	0.96	0.16	0.20	0.43
0.50	1000	0.94	1.04	2.07	1.05	1.03	1.04	0.25	0.27	0.64
0.00	600	-0.15	-0.09	-0.16	1.09	1.06	1.02	0.05	0.05	0.04
0.00	1000	-0.09	-0.05	-0.04	1.07	1.09	1.06	0.06	0.06	0.07
-0.50	600	-0.41	-0.32	-0.50	1.06	1.05	1.00	0.03	0.04	0.02
-0.50	1000	-0.41	-0.38	-0.59	1.05	1.04	1.03	0.03	0.03	0.01
-1.00	600	-0.30	-0.18	-0.27	1.06	1.13	1.03	0.03	0.06	0.03
-1.00	1000	-0.25	-0.22	-0.30	1.07	1.01	0.99	0.03	0.04	0.03

Table 26

Results under Model 2b, with $\pi(\mathbf{X}_i) = exp(-0.3 + 0.2X_{1i} + 0.6X_{5i})/\{1 + exp(-0.3 + 0.2X_{1i} + 0.6X_{5i})\}$ and $L = 42$. Mean (μ), standard deviation (σ), and power or type I error ($1 - \beta$ /α) of the one-step value difference test statistic is based on 500 simulated data sets of $n = \{600, 1000\}$. Subscripts "IPW", "AIPW", and "CAIPW" specify whether the results are baesd on T_{IPW}, T_{AIPW}, or T_{CAIPW}, respectively. Largest standard error for μ, σ, and $1 - \beta$ /α is .05 and 0.08, and 0.02, respectively.

c	n	μ_{IPW}	μ_{AIPW}	μ_{CAIPW}	σ_{IPW}	σ_{AIPW}	σ_{CAIPW}	$1 - \beta_{\text{IPW}}$ /α_{IPW}	$1 - \beta_{\text{AIPW}}$ /α_{AIPW}	$1 - \beta_{\text{CAIPW}}$ /α_{CAIPW}
1.25	600	2.41	2.57	4.22	1.05	0.99	0.99	0.76	0.82	1.00
1.25	1000	3.28	3.47	5.80	1.02	1.03	1.05	0.95	0.97	1.00
0.75	600	1.47	1.60	2.52	1.05	1.01	0.98	0.41	0.49	0.84
0.75	1000	2.06	2.22	3.55	1.02	1.04	1.06	0.66	0.72	0.97
0.50	600	0.87	1.00	1.57	1.05	1.01	1.00	0.23	0.23	0.47
0.50	1000	1.32	1.46	2.31	1.03	1.06	1.07	0.38	0.43	0.75
0.00	600	-0.18	-0.12	-0.16	1.07	1.06	0.98	0.04	0.05	0.04
0.00	1000	-0.14	-0.10	-0.08	1.03	1.05	1.03	0.05	0.06	0.05
-0.50	600	-0.47	-0.38	-0.50	1.01	1.04	0.99	0.01	0.04	0.01
-0.50	1000	-0.47	-0.43	-0.56	1.02	1.05	1.02	0.02	0.03	0.02
-1.00	600	-0.36	-0.26	-0.31	1.01	1.10	1.07	0.02	0.05	0.03
-1.00	1000	-0.24	-0.20	-0.26	1.04	1.07	1.02	0.05	0.05	0.03

Table 27

Results under Model 2c, with $\pi(\mathbf{X}_i) = exp(-0.3 + 0.2X_{1i} + 0.6X_{5i})/\{1 + exp(-0.3 + 0.2X_{1i} + 0.6X_{5i})\}$ *and* $L = 33$. *Mean (μ), standard deviation (σ), and power or type I error* $(1 - \beta\ /\alpha)$ *of the one-step value difference test statistic is based on 500 simulated data sets of* $n = \{600, 1000\}$. *Subscripts "IPW", "AIPW", and "CAIPW" specify whether the results are baesd on* T_{IPW}, T_{AIPW}, *or* T_{CAIPW}, *respectively. Largest standard error for* μ, σ, *and* $1 - \beta\ /\alpha$ *is* .05 *and* 0.08, *and* 0.02, *respectively.*

c	n	μ_{IPW}	μ_{AIPW}	μ_{CAIPW}	σ_{IPW}	σ_{AIPW}	σ_{CAIPW}	$1-\beta_{\text{IPW}}/\alpha_{\text{IPW}}$	$1-\beta_{\text{AIPW}}/\alpha_{\text{AIPW}}$	$1-\beta_{\text{CAIPW}}/\alpha_{\text{CAIPW}}$
1.25	600	1.77	1.88	3.74	1.12	1.11	1.02	0.57	0.57	0.99
1.25	1000	2.36	2.49	5.19	0.96	0.98	1.03	0.77	0.80	1.00
0.75	600	1.09	1.19	2.24	1.12	1.11	1.04	0.31	0.34	0.72
0.75	1000	1.50	1.62	3.23	0.96	0.98	1.05	0.45	0.49	0.94
0.50	600	0.68	0.79	1.40	1.11	1.11	1.07	0.19	0.23	0.40
0.50	1000	0.98	1.09	2.10	0.95	0.97	1.06	0.24	0.29	0.66
0.00	600	-0.16	-0.10	-0.17	1.12	1.11	1.00	0.04	0.05	0.02
0.00	1000	-0.14	-0.08	-0.03	0.96	1.01	1.02	0.04	0.04	0.05
-0.50	600	-0.41	-0.34	-0.54	1.11	1.14	1.10	0.03	0.04	0.02
-0.50	1000	-0.44	-0.40	-0.55	0.99	0.97	1.01	0.02	0.02	0.02
-1.00	600	-0.33	-0.23	-0.32	1.07	1.07	1.04	0.03	0.04	0.04
-1.00	1000	-0.26	-0.22	-0.27	1.03	1.00	0.99	0.04	0.03	0.03

Table 28
Results from application to ACTG175 data

Treatment	Method	Test Statistic	P-value
zidovudine + didanosine	$T_{\text{IPW,SAP-match}}$	3.93	< 0.01
zidovudine + didanosine	$T_{\text{IPW,SBT}}$	0.97	0.17
zidovudine + didanosine	$T_{\text{AIPW,SAP-match}}$	3.68	< 0.01
zidovudine + didanosine	$T_{\text{AIPW,SBT}}$	2.08	0.02
zidovudine + zalcitabine	$T_{\text{IPW,SAP-match}}$	-1.45	0.93
zidovudine + zalcitabine	$T_{\text{IPW,SBT}}$	-0.63	0.73
zidovudine + zalcitabine	$T_{\text{AIPW,SAP-match}}$	-0.52	0.70
zidovudine + zalcitabine	$T_{\text{AIPW,SBT}}$	-1.98	0.98

Table 29

Results are based on 500 simulated data sets. Both an uncensored event setting (Outcome labeled 'Uncensored') and a censored event setting (Outcome labeled 'Censored') from the paper are considered. Data for the uncensored setting were generated according to the Model 1 scheme 1 observational study design described in Section 5.1 of the paper, with $c = 0.3$. *The test statistic used to calculate power in the uncensored setting is* $T_{AIPW,\ SBT}$. *Data for the censored setting were generated according to Model 1b with* $\pi(\mathbf{X}_i) = exp(-0.3 + 0.2X_{1i} + 0.6X_{5i})/\{1 + exp(-0.3 + 0.2X_{1i} + 0.6X_{5i})\}$, $L = 42$ *and* $c = 0.5$. *The test statistic used to calculate power in the censored setting is* $T^S_{CAIPW,\ SBT}$. *Largest standard error for power is* 0.021.

Outcome	n	m	Power
Uncensored	200	2	0.16
Uncensored	200	5	0.18
Uncensored	200	10	0.16
Uncensored	200	20	0.17
Uncensored	1000	2	0.78
Uncensored	1000	5	0.77
Uncensored	1000	10	0.76
Uncensored	1000	20	0.79
Censored	200	2	0.32
Censored	200	5	0.30
Censored	200	10	0.32
Censored	200	20	0.31
Censored	600	2	0.78
Censored	600	5	0.75
Censored	600	10	0.78
Censored	600	20	0.78
