# Supplementary material for: A general framework for subgroup detection via one-step value difference estimation
Source: Biometrics. Author manuscript; Available in PMC 2023 Dec 4. (PMC10694635; doi:10.1111/biom.13711)
Supplement: code [file NIHMS1945184-supplement-code.zip › Rcode/Censored_outcome/README copy.rtf]

Author: Dana JohnsonFunction Files:time_to_event_functions.R - Contains the functions required to run the randomized controlled trial (RCT) and observational simulations under the various combinations of failure time model and censoring time model discussed in Section 5.2 of the paper.                                             Simulation Files:Randomized Controlled Trial (RCT):survival_IPW_5050_scenario1a.R 	•Imports time_to_event_functions.R	•Performs RCT simulations under model 1a and the IPW, SBT version of the test statistic (T_{IPW, SBT} from the paper).survival_AIPW_5050_scenario1a.R 	•Imports time_to_event_functions.R	•Performs RCT simulations under model 1a and the AIPW, SBT version of the test statistic (T_{AIPW, SBT} from the paper).survival_CAIPW_5050_scenario1a.R 	•Imports time_to_event_functions.R	•Performs RCT simulations under model 1a and the CAIPW, SBT version of the test statistic (T_{CAIPW, SBT} from the paper).survival_IPW_5050_scenario1b.R 	•Imports time_to_event_functions.R	•Performs RCT simulations under model 1b and the IPW, SBT version of the test statistic (T_{IPW, SBT} from the paper).survival_AIPW_5050_scenario1b.R 	•Imports time_to_event_functions.R	•Performs RCT simulations under model 1b and the AIPW, SBT version of the test statistic (T_{AIPW, SBT} from the paper).survival_CAIPW_5050_scenario1b.R 	•Imports time_to_event_functions.R	•Performs RCT simulations under model 1b and the CAIPW, SBT version of the test statistic (T_{CAIPW, SBT} from the paper).survival_IPW_5050_scenario1c.R 	•Imports time_to_event_functions.R	•Performs RCT simulations under model 1c and the IPW, SBT version of the test statistic (T_{IPW, SBT} from the paper).survival_AIPW_5050_scenario1c.R 	•Imports time_to_event_functions.R	•Performs RCT simulations under model 1c and the AIPW, SBT version of the test statistic (T_{AIPW, SBT} from the paper).survival_CAIPW_5050_scenario1c.R 	•Imports time_to_event_functions.R	•Performs RCT simulations under model 1c and the CAIPW, SBT version of the test statistic (T_{CAIPW, SBT} from the paper).survival_IPW_5050_scenario2a.R 	•Imports time_to_event_functions.R	•Performs RCT simulations under model 2a and the IPW, SBT version of the test statistic (T_{IPW, SBT} from the paper).survival_AIPW_5050_scenario2a.R 	•Imports time_to_event_functions.R	•Performs RCT simulations under model 2a and the AIPW, SBT version of the test statistic (T_{AIPW, SBT} from the paper).survival_CAIPW_5050_scenario2a.R 	•Imports time_to_event_functions.R	•Performs RCT simulations under model 2a and the CAIPW, SBT version of the test statistic (T_{CAIPW, SBT} from the paper).survival_IPW_5050_scenario2b.R 	•Imports time_to_event_functions.R	•Performs RCT simulations under model 2b and the IPW, SBT version of the test statistic (T_{IPW, SBT} from the paper).survival_AIPW_5050_scenario2b.R 	•Imports time_to_event_functions.R	•Performs RCT simulations under model 2b and the AIPW, SBT version of the test statistic (T_{AIPW, SBT} from the paper).survival_CAIPW_5050_scenario2b.R 	•Imports time_to_event_functions.R	•Performs RCT simulations under model 2b and the CAIPW, SBT version of the test statistic (T_{CAIPW, SBT} from the paper).survival_IPW_5050_scenario2c.R 	•Imports time_to_event_functions.R	•Performs RCT simulations under model 2c and the IPW, SBT version of the test statistic (T_{IPW, SBT} from the paper).survival_AIPW_5050_scenario2c.R 	•Imports time_to_event_functions.R	•Performs RCT simulations under model 2c and the AIPW, SBT version of the test statistic (T_{AIPW, SBT} from the paper).survival_CAIPW_5050_scenario2c.R 	•Imports time_to_event_functions.R	•Performs RCT simulations under model 2c and the CAIPW, SBT version of the test statistic (T_{CAIPW, SBT} from the paper).Observational Study:obs_survival_IPW_5050_scenario1a.R	•Imports time_to_event_functions.R	•Performs observational study simulations under model 1a and the IPW, SBT version of the test statistic (T_{IPW, SBT} from the paper).obs_survival_AIPW_5050_scenario1a.R	•Imports time_to_event_functions.R	•Performs observational study simulations under model 1a and the AIPW, SBT version of the test statistic (T_{AIPW, SBT} from the paper).obs_survival_CAIPW_5050_scenario1a.R	•Imports time_to_event_functions.R	•Performs observational study simulations under model 1a and the CAIPW, SBT version of the test statistic (T_{CAIPW, SBT} from the paper).obs_survival_IPW_5050_scenario1b.R	•Imports time_to_event_functions.R	•Performs observational study simulations under model 1b and the IPW, SBT version of the test statistic (T_{IPW, SBT} from the paper).obs_survival_AIPW_5050_scenario1b.R	•Imports time_to_event_functions.R	•Performs observational study simulations under model 1b and the AIPW, SBT version of the test statistic (T_{AIPW, SBT} from the paper).obs_survival_CAIPW_5050_scenario1b.R	•Imports time_to_event_functions.R	•Performs observational study simulations under model 1b and the CAIPW, SBT version of the test statistic (T_{CAIPW, SBT} from the paper).obs_survival_IPW_5050_scenario1c.R	•Imports time_to_event_functions.R	•Performs observational study simulations under model 1c and the IPW, SBT version of the test statistic (T_{IPW, SBT} from the paper).obs_survival_AIPW_5050_scenario1c.R	•Imports time_to_event_functions.R	•Performs observational study simulations under model 1c and the AIPW, SBT version of the test statistic (T_{AIPW, SBT} from the paper).obs_survival_CAIPW_5050_scenario1c.R	•Imports time_to_event_functions.R	•Performs observational study simulations under model 1c and the CAIPW, SBT version of the test statistic (T_{CAIPW, SBT} from the paper).obs_survival_IPW_5050_scenario2a.R	•Imports time_to_event_functions.R	•Performs observational study simulations under model 2a and the IPW, SBT version of the test statistic (T_{IPW, SBT} from the paper).obs_survival_AIPW_5050_scenario2a.R	•Imports time_to_event_functions.R	•Performs observational study simulations under model 2a and the AIPW, SBT version of the test statistic (T_{AIPW, SBT} from the paper).obs_survival_CAIPW_5050_scenario2a.R	•Imports time_to_event_functions.R	•Performs observational study simulations under model 2a and the CAIPW, SBT version of the test statistic (T_{CAIPW, SBT} from the paper).obs_survival_IPW_5050_scenario2b.R	•Imports time_to_event_functions.R	•Performs observational study simulations under model 2b and the IPW, SBT version of the test statistic (T_{IPW, SBT} from the paper).obs_survival_AIPW_5050_scenario2b.R	•Imports time_to_event_functions.R	•Performs observational study simulations under model 2b and the AIPW, SBT version of the test statistic (T_{AIPW, SBT} from the paper).obs_survival_CAIPW_5050_scenario2b.R	•Imports time_to_event_functions.R	•Performs observational study simulations under model 2b and the CAIPW, SBT version of the test statistic (T_{CAIPW, SBT} from the paper).obs_survival_IPW_5050_scenario2c.R	•Imports time_to_event_functions.R	•Performs observational study simulations under model 2c and the IPW, SBT version of the test statistic (T_{IPW, SBT} from the paper).obs_survival_AIPW_5050_scenario2c.R	•Imports time_to_event_functions.R	•Performs observational study simulations under model 2c and the AIPW, SBT version of the test statistic (T_{AIPW, SBT} from the paper).obs_survival_CAIPW_5050_scenario2c.R	•Imports time_to_event_functions.R	•Performs observational study simulations under model 2c and the CAIPW, SBT version of the test statistic (T_{CAIPW, SBT} from the paper).
