# Supplementary material for: A general framework for subgroup detection via one-step value difference estimation
Source: Biometrics. Author manuscript; Available in PMC 2023 Dec 4. (PMC10694635; doi:10.1111/biom.13711)
Supplement: code [file NIHMS1945184-supplement-code.zip › Rcode/Uncensored_outcome/README.rtf]

Author: Dana JohnsonFunction Files:model1_rct_ipw_sapmatch_functions.R -  Contains the functions required to run the randomized controlled trial (RCT) simulations under model 1 and the IPW,SAP-match version of the test statistic (T_{IPW, SAP-match} from the paper).model1_rct_ipw_sbt_functions.R - Contains the functions required to run the RCT simulations under model 1 and the IPW,SBT version of the test statistic (T_{IPW,SBT} from the paper).model1_rct_aipw_sapmatch_functions.R - Contains the functions required to run the RCT simulations under model 1 and the AIPW,SAP-match version of the test statistic (T_{AIPW, SAP-match} from the paper).model1_rct_aipw_sbt_functions.R  - Contains the functions required to run the RCT simulations under model 1 and the AIPW,SBT version of the test statistic (T_{AIPW, SBT} from the paper).model1_obs_pi5_ipw_sapmatch_functions.R - Contains the functions required to run the observational study simulations under model 1, beta = (-0.3, 0.2, 0.6)^T,  and the IPW,SAP-match version of the test statistic (T_{IPW, SAP-match} from the paper). Recall that when beta = (-0.3, 0.2, 0.6)^T, the marginal treatment probability is approximately 0.5.model1_obs_pi5_ipw_sbt_functions.R -  Contains the functions required to run the observational study simulations under model 1, beta = (-0.3, 0.2, 0.6)^T,  and the IPW,SBT version of the test statistic (T_{IPW, SBT} from the paper). Recall that when beta = (-0.3, 0.2, 0.6)^T, the marginal treatment probability is approximately 0.5.model1_obs_pi5_aipw_sapmatch_functions.R -  Contains the functions required to run the observational study simulations under model 1, beta = (-0.3, 0.2, 0.6)^T,  and the AIPW,SAP-match version of the test statistic (T_{AIPW, SAP-match} from the paper). Recall that when beta = (-0.3, 0.2, 0.6)^T, the marginal treatment probability is approximately 0.5.model1_obs_pi5_aipw_sbt_funcions.R -  Contains the functions required to run the observational study simulations under model 1, beta = (-0.3, 0.2, 0.6)^T,  and the AIPW,SBT version of the test statistic (T_{AIPW, SBT} from the paper). Recall that when beta = (-0.3, 0.2, 0.6)^T, the marginal treatment probability is approximately 0.5.Simulation Files:Randomized Controlled Trial (RCT):model1_rct_ipw_sapmatch.R 	•Imports model1_rct_ipw_sapmatch_functions.R	•Performs RCT simulations under model 1 and the IPW,SAP-match version of the test statistic (T_{IPW, SAP-match} from the paper).model1_rct_ipw_sbt.R	•Imports model1_rct_ipw_sbt_functions.R	•Performs RCT simulations under model 1 and the IPW,SBT version of the test statistic (T_{IPW, SBT} from the paper).model1_rct_aipw_sapmatch.R	•Imports model1_rct_aipw_sapmatch_functions.R	•Performs RCT simulations under model 1 and the AIPW,SAP-match version of the test statistic (T_{AIPW, SAP-match} from the paper).model1_rct_aipw_sbt.R	•Imports model1_rct_aipw_sbt_functions.R	•Performs RCT simulations under model 1 and the AIPW,SBT version of the test statistic (T_{AIPW, SBT} from the paper).Observational Study:model1_obs_pi5_ipw_sapmatch.R	•Imports model1_obs_pi5_ipw_sapmatch_functions.R	•Performs observational study simulations under model 1, beta = (-0.3, 0.2, 0.6)^T, and the IPW,SAP-match version of the test statistic (T_{IPW, SAP-match} from the paper). Recall that when beta = (-0.3, 0.2, 0.6)^T, the marginal treatment probability is approximately 0.5.model1_obs_pi5_ipw_sbt.R	•Imports model1_obs_pi5_ipw_sbt_functions.R	•Performs observational study simulations under model 1, beta = (-0.3, 0.2, 0.6)^T,  and the IPW,SBT version of the test statistic (T_{IPW, SBT} from the paper). Recall that when beta = (-0.3, 0.2, 0.6)^T, the marginal treatment probability is approximately 0.5.model1_obs_pi5_aipw_sapmatch.R	•Imports model1_obs_pi5_aipw_sapmatch_functions.R	•Performs observational study simulations under model 1, beta = (-0.3, 0.2, 0.6)^T, and the AIPW,SAP-match version of the test statistic (T_{AIPW, SAP-match} from the paper). Recall that when beta = (-0.3, 0.2, 0.6)^T, the marginal treatment probability is approximately 0.5.model1_obs_pi5_aipw_sbt.R	•Imports model1_obs_pi5_aipw_sbt_functions.R	•Performs observational study simulations under model 1, beta = (-0.3, 0.2, 0.6)^T,  and the AIPW,SBT version of the test statistic (T_{AIPW, SBT} from the paper). Recall that when beta = (-0.3, 0.2, 0.6)^T, the marginal treatment probability is approximately 0.5.
